# Determination of Molecular Interactions between Copper Pigments and Soybean Oil Alkyd Resins in Paints: A Spectroscopic Study

**DOI:** 10.1021/acsomega.5c05576

**Published:** 2026-05-07

**Authors:** Thiago Guimarães Costa, António José Estêvão Grande Candeias, Enzo Gimenes Gazotto, Feik Amil de Campos Júnior, Leonardo Negri Furini, Adolfo Horn

**Affiliations:** † Laboratory of Materials, Atelier for Conservation and Restoration of Cultural Heritage, Santa Catarina State Culture Foundation, 88025-200 Florianópolis, SC, Brazil; ‡ HERCULES Laboratory, Institute for Advanced Studies and Research, University of Évora, Largo Marquês de Marialva 8, Évora 7000-809, Portugal; § Department of Chemistry, Federal University of Santa Catarina (UFSC), 88040-900 Florianópolis, SC, Brazil; ∥ Department of Physics, Federal University of Santa Catarina (UFSC), 88040-900 Florianópolis, SC, Brazil

## Abstract

Alkyd paints are widely used in modern painting and can be obtained commercially or prepared from pure materials. Various formulations can be used to obtain these paints. Copper pigments can be found in various works of art and in industrial paints. The use of these pigments in modern resins can be a low-cost alternative for different applications. However, studies must be conducted to ensure safety when applying these paints. In this work, the interaction of Cu­(II) present in malachite, azurite, and copper acetate pigments with soybean oil-based alkyd resins when subjected to heat treatment above 50 °C was characterized. The results show that paintings containing malachite and azurite exhibited colorimetric Δ*E* values of 2.77 and 3.72, respectively, showing no significant chromatic change during the studied heat period. Fourier transform infrared (FTIR) spectroscopy revealed a decrease in the intensity of ester and phthalic groups in the resin over time, indicating that reactions occurred in these groups during the sample aging process. Raw copper acetate was initially present in the dimeric form; however, when mixed with a binder, monomeric species were formed, and the intensity of dimers decreased with the aging period, leading to the formation of only monomeric species with a color change. Scanning electron microscopy (SEM) analysis showed that the paint layers remained uniform even after the studied aging period. μ-Raman showed signals below 500 cm^–1^, which are attributed to Cu–O vibrational modes of new structures formed during the aging process of the paints. The chemical transformations do not lead to color changes in the pigments of malachite and azurite, so the results suggest that soybean oil-based alkyd resin could be a viable and cost-effective alternative to be used in the formulation with copper pigments. Furthermore, the data present can help understand the behavior of alkyd paints in the presence of copper pigments, assisting in the advancement of materials for use in painting materials.

## Graphical abstract

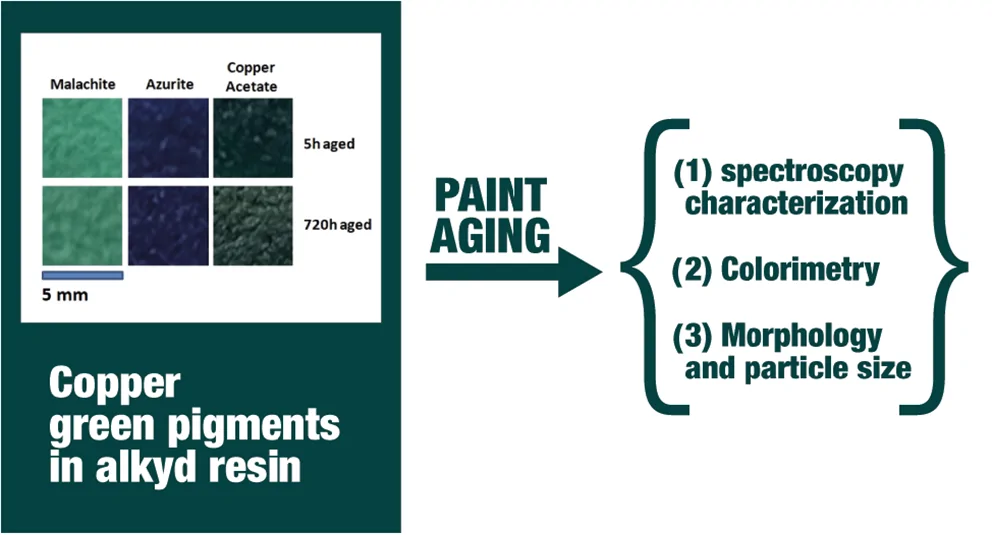

## 1 Introduction

Alkyd resins are synthetic polyesters modified with fatty acids, which are prepared by the condensation polymerization of polyols, polyacids, and vegetable oils. Their high compatibility with a variety of polymers, combined with their solubility in organic solvents or water, has secured their widespread application in paints, coatings, and conservation materials.^1,2^ A wide variety of vegetable oils can be used in the formulation of alkyd resins, but the quantity of other reagents, such as polyols and polyacids, which originate from nonrenewable sources, is affected by the proportion of oil incorporated into the resin composition. The physical and chemical properties of the resulting paint are directly influenced by the constitution of the resin.^3^

Alkyd resins have played a significant role in art. Since their introduction in the 1930s as an evolution of oil paints, artists such as Jackson Pollock and Pablo Picasso adopted these resins in their works due to their fast drying time, lower cost, and versatility in different formulations.^4^ In Brazil, for example, alkyd resin became of great artistic interest starting in the 1950s, when the properties of this resin began to be experimented with in artworks, such as its gloss, durability, and especially its much faster drying time compared to other binders used at the time. Ivan Serpa and Waldemar Cordeiro are examples of Brazilian artists who used alkyd resins during this period.^5,6^

Brazil is one of the largest producers of soybean oil in the world, and soybean oil-based alkyd resins are produced and commercialized in the country. Since it can be reused, the use of this oil is an innovative solution that combines economic efficiency with environmental responsibility for the production of alkyd paints.^7^

The aging of alkyd resins is a complex phenomenon that involves a series of chemical reactions occurring over time, especially when they are exposed to environmental conditions. This process, which directly affects the mechanical and chemical properties of materials, is influenced by factors such as atmospheric oxygen, ultraviolet light, heat, humidity, and environmental pollutants.^8–10^

The interaction with inorganic pigments present in paints can lead to the formation of degradation products harmful to artworks. Additionally, the aging and degradation process of the resin can show variations depending on different inorganic pigments. Duce et al.^11^ demonstrated that the interaction between organic pigments with alkyd resins is considerably strong, while inorganic pigments appear to be more stable. Michèlle Gunn et al. showed the capacity of linoleic and abietic acids to extract copper­(II) ions from copper acetate.^12^ Recently, Punis et al.^13^ demonstrated the interactions between malachite and azurite pigments with fatty acids derived from aged oils in paintings using electron paramagnetic resonance (EPR) spectroscopy, resulting in the formation of metal soaps. The authors also conclude that the nature of these copper complexes can significantly vary depending on the organic binder used, and different binders exhibit variable affinities toward copper ions. Alter et al.^14^ reported the darkening of oil paintings in the presence of pigments derived from copper acetate. In this way, the interaction between aged alkyd resins and copper pigments are unknow. Kazarian et al.^15^ report the use of Fourier transform infrared (FTIR) techniques for characterizing chemical modifications in oil paints and the formation of metallic soaps.

Alkyd resins are currently commercially available, and the search for new formulations of biobased coatings is constant, as reported in the literature.^16^ Therefore, it is important to investigate the interaction mechanism of this resin with inorganic pigments, since the inorganic pigments can affect the stability of the resin.^11^

Currently, there are no commercially available formulations of alkyd resins that contain copper pigments. This absence is likely due to the effects of the resins on the pigments. For instance, Duce et al.^11^ reported that the copper phthalocyanine pigments, known as phthalo blue (PB15) and chlorinated copper phthalocyanine, referred to as phthalo green (PG7), lose their color when subjected to an accelerated aging process involving exposure to an acetic acid atmosphere. Recently, Poli et al.^16^ also indicated that copper-based pigments can be involved in the formation of metal soaps in the presence of alkyd resins. Such data prompt us to investigate whether malachite, azurite, and copper acetate pigments exhibit similar behaviors to those cited above. By examining the interactions of soybean oil-based alkyd paints on canvas with malachite, azurite, and copper acetate pigments during accelerated thermal treatment, using advanced spectroscopic and colorimetric methods, this work aimed to clarify the interaction between alkyd resin and the Cu­(II) pigments, potentially leading to innovative formulations.

## 2 Experimental Section

### 2.1 Preparation of Mockup Samples and Accelerated Aging Process

Paint mockups were prepared by manually mixing a commercial soybean oil-based alkyd resin (Soy Alkyd Medium Resin, WEG, Brazil) with the pigments azurite (Cu_3_(CO_3_)_2_(OH)_2_, Kremer Pigments, Germany), malachite (Cu_2_(OH)_2_CO_3_, Vetec, Brazil), and copper­(II) acetate (Cu_2_(OAc)_4_·H_2_O, Sigma-Aldrich) in a pigment-to-binder weight ratio of 4:1. The mixtures were homogeneously applied onto linen canvases (area approximately 25 cm^2^) using a brush and subjected to heat treatment.

Accelerated thermal aging was conducted in an oven maintained at 50 °C, a temperature chosen to promote oxidative and thermal degradation processes without inducing melting of the main fatty acids present in the glycerides formed upon hydrolysis and oxidation.^17^ Samples were submitted to thermal treatment for up to 720 h. A summary of the materials employed is provided in Table 1.

**Table 1:** Description of the Alkyd Binder and the Copper Pigments

binder	chemical composition	commercial name
alkyd resin	medium-chain polymer soy oil-modified polyester-resin based on phthalic anhydride and glycerol	Soy Alkyd Medium Resin (WEG, Brazil)

pigment	color index	chemical composition
azurite	PB30	Cu_2_(CO_3_)_2_(OH)_2_
malachite	PG39	Cu_2_(OH)_2_CO_3_
copper(II) acetate	PG20	Cu_2_(OAc)_4_·H_2_O

All paint samples were air-dried under dark conditions at 25 ± 3 °C and 55 ± 5% RH for a minimum of 720 h to obtain stable polychrome films suitable for further tests and comparison with the standard, fresh, and thermally cured paint samples. The pure alkyd resin was also subjected to thermal treatment for comparison with the paints. In this work, the use of “fresh samples” indicates that the analyses were performed immediately after the dye or pure resin was applied to the linen.

### 2.2 Colorimetry

Colorimetric measurements were performed using a portable colorimeter equipped with the Color Grab application, calibrated with a white standard. Measurements were acquired at five distinct points per sample (center and corners) using a 3 mm diameter measurement area.^18,19^ The color parameters L*, a*, and b* were recorded in the CIELab color space system following the Commission Internationale de l′Éclairage (CIE) standards.^18,19^ The total color difference (Δ*E*) was calculated according to eq 1:

1 $$\Delta E={\left[{\left(\Delta L\right)}^{*2}+{\left(\Delta a\right)}^{*2}+{\left(\Delta b\right)}^{*2}\right]}^{1/2}$$

where ΔL*, Δa*, and Δb* represent the changes in brightness, green–red, and blue–yellow chromatic axes, respectively. A Δ*E* below 5 is generally considered imperceptible or minimally perceptible to the human eye. Above 5 units, observers perceive two different colors.^19–21^

### 2.3 Fourier Transform Infrared (FTIR) Spectroscopy

Infrared spectra were collected using a JASCO FTIR-4100 spectrometer with a resolution of 4 cm^–1^, utilizing an attenuated total reflectance (ATR) accessory with a ZnSe crystal. The canvas paintings samples were analyzed directly on the ATR crystal, and all spectra were recorded with 64 scans. For comparison purposes, all spectra were normalized between 0 and 1 based on minimum and maximum intensities. Spectral assignments focused on characteristic absorptions of ester, carboxylate, and phthalic anhydride groups, as well as pigment-related bands.

### 2.4 EPR Spectroscopy

Electron paramagnetic resonance (EPR) analysis was performed with a Bruker EMX spectrometer operating at ∼9.4 GHz, with a 10 G modulation amplitude, 100 kHz modulation frequency, and 5000 G (500 mT) field scan at 291 K. The spectra and *g*-factor values were obtained using Bruker Xenon software. The *g* values were corrected before the simulations using a (MgO:Cr^3+^) standard with *g* = 1.9797 and can be observed in each collected spectrum.^22^

### 2.5 SEM-Energy-Dispersive X-ray Spectroscopy (EDS)

The linen painting samples were cut and fixed with carbon tape on stubs. Each sample was placed on a different stub and then blown with compressed air. Micrographs were collected using a Phenom-X scanning electron microscope under the following analysis conditions: acceleration voltage: 10 kV, vacuum: low (60 Pa), spot size: image, magnifications: 500× and 1000×, and detector: backscattered electron (BSD).

Five pairs of images were collected from thermal treatment samples at 5 and 720 h for comparison. Each image pair was taken from a different sample point: four near the corners and one near the center. The acquisition time was 1 s, in medium quality, with a scan size of 1920 × 1200. The pigment size distribution within the paint layers was determined using the ImageJ software.^23^

### 2.6 Raman Spectroscopy

Raman spectra were recorded using a Renishaw inVia spectrometer coupled with a confocal optical microscope (Leica DM2700M). The laser line was employed at 532 nm to obtain a better signal/noise ratio. Also, the laser power (500 mW) was adjusted accordingly to avoid burning and focusing the laser beam on the sample. The spectra were collected under a 10 s exposure time per scan and accumulated 10 times. Measurements were made in direct-contact mode with the samples placed on glass slides. Key vibrational bands for copper pigments and binder degradation products were identified on the basis of previously published spectral assignments. All of the spectra were submitted to baseline correction using the Wire 5.6 software package.

## 3 Result and Discussion

### 3.1 Colorimetry

The variations in a*, b*, L*, and total *E* for the fresh and 720 h thermal treatment paint samples are presented in Figure 1. Full data sets for intermediate aging times are provided in Figure S1 and Table S1 (Supporting Information). The results presented in Figure 1 show considerably smaller variations in Δ*E* for the paints containing malachite and azurite, 2.77 and 3.72, respectively. It is known that variations of less than 5 units are hardly noticeable for most observers.^18^ However, there is a slight shift toward red tones of azurite with Δ*a* of 3.1 and the slight darkening of malachite with a Δ*L* value of −2.4. However, copper acetate showed large colorimetric variations, both in the L* and b* parameters, with variations of 9.8 and −5.3, respectively. A positive ΔL* indicates the lightening of the sample, while a negative Δb* reveals a change toward blue.

**Figure 1:**
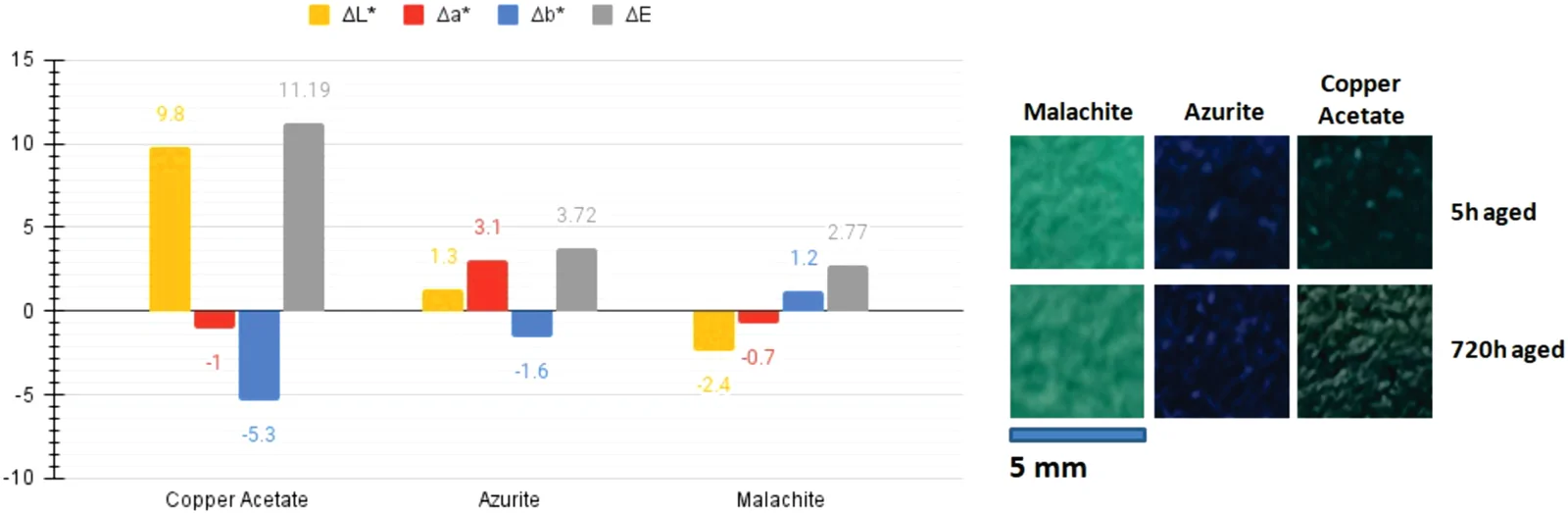
ΔL*, Δa*, Δb*, and Δ*E* values for paints containing malachite, azurite, and copper acetate after 720 h of thermal aging and microphotography of the paints in 5 h and 720 h thermal treatment.

The results show that the changes in the colorimetric parameters of malachite and azurite were considerably smaller when compared to copper acetate, revealing the high resilience to color changes during the heat treatment of alkyd binder in paint. Our results corroborate the studies conducted by Zuo et al.^24^ that recently demonstrate the high resistance to color change presented by malachite in oil when exposed to thermal aging, presenting a low Δ*E*.

In general, the aging of oil resins and their derivatives generates compounds such as aldehydes and carboxylic acids, which influence the optical properties of the resins, varying the luminosity (L*) and altering shades of green–red (a*) and blue–yellow (b*).^8,24^

The significant changes in the Δ*L* and Δ*E* observed for copper acetate indicate its instability in organic environments and tendency to chemical degradation. Alter et al.^14^ also reported chromatic alteration in copper acetate in historical paintings containing oily binders. On the other hand, azurite and malachite showed higher stability, as described previously.^25–28^

It has been reported that during the degradation of copper acetate, products such as basic acetates and copper hydroxides are formed, with copper hydroxide being one of the main causes of the color change from bluish-green to lighter shades of blue, proving to be the main agent responsible for the decrease in Δb* and an increase in ΔL* during the aging of the samples.^24–27,29,30^ Aiming to understand such behavior from a molecular point of view, we used spectroscopic methods to characterize the degradation products of copper acetate in soybean oil alkyd resins.

### 3.2 FTIR Spectroscopy

The FTIR spectra of thermally treated alkyd paints containing malachite, azurite, and copper acetate can be seen in Figure S2. The spectra of the standard materials used in the work are presented in Figure S3, and the characteristic vibrational frequencies are presented in Table S2. The spectra of malachite and azurite paints show the same behavior during accelerated aging. Regarding the resin, there is a considerable decrease in the intensity of the bands at 2852 and 2922 cm^–1^ due to the polymerization of aliphatic C–H bonds caused by the oxidation of double bonds during the alkyd resin drying.^31–33^

Figure 2A–C shows the superimposed spectra with normalized intensity between 1800 and 900 cm^–1^. It can be observed that the ester carbonyl bands present around 1721 cm^–1^ undergo a decrease in intensity, attributed to changes in the chemical environment of the carbonyl groups, associated with oxidation and cross-linking reactions occurring during the curing of the alkyd network and formation of other functional groups^31–33^ (Figure S4 with more details), similar modifications are indeed observed also when comparing with the dark paint samples in museum conditions (Figure S6). Still in relation to the ester, a decrease in the intensity of the νC–O–C stretching at 1260 cm^–1^ is also observed.

**Figure 2:**
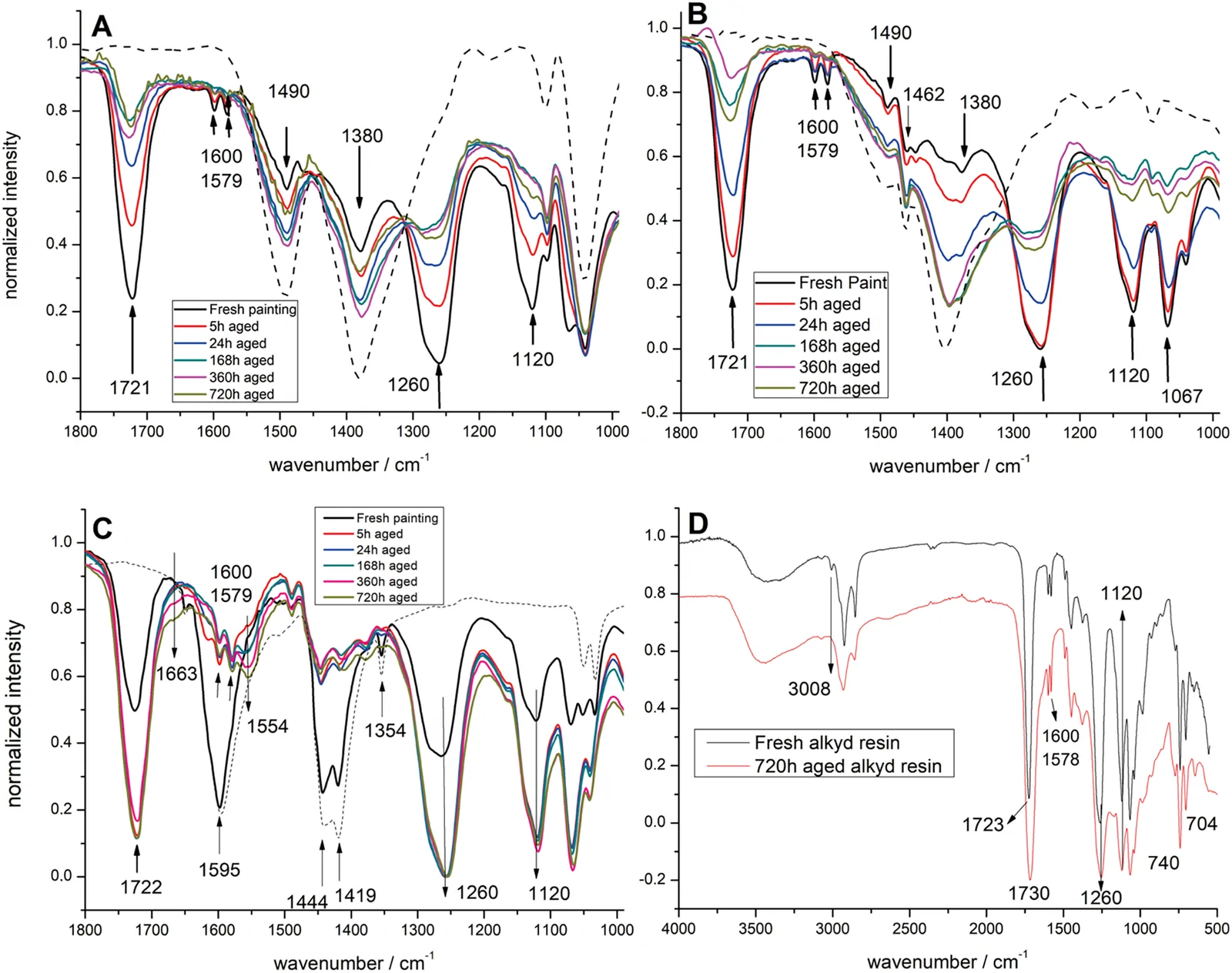
Intensity-normalized FTIR spectra of thermal treatment soybean resin alkyd paints containing malachite (A), azurite (B), and copper acetate (C) pigments in the 1800–990 cm^–1^ region and (D) fresh and thermally treated alkyd resin for comparison. Dashed lines correspond to raw standard pigments.

At 1579 and 1600 cm^–1^, two bands can be observed that represent stretching of double bonds between carbons of the aromatic ring of phthalic anhydride, which decrease in intensity with increasing aging time, showing that there are reactions that occur in the anhydride group present in the structure of the alkyd resin. The same behavior is observed in alkyd resins in the work of Pagnin et al.^8^

In the spectra of the three systems, bands are observed at 1120 and 1067 cm^–1^. Duce et al.^1^ report that such stretching can be attributed to the νC–H, νC–C, and νC–O bonds of the alkyd resin. In both spectra, a decrease in the intensity of these stretching is observed as a function of the increase in aging time, indicating chemical transformations during the curing of the resin. In the spectrum of the copper acetate/resin system, only after 360 h of aging does a decrease in the intensity of these bands occur.

The band that represents vibrations of δ C–H outside the aromatic plane (704 cm^–1^), as well as at 740 cm^–1^,^31–33^ shows a decrease in intensity with increasing aging time, resulting in the total absence of the band after 168 h, where it is possible to observe the presence of a shoulder, which becomes more evident at 720 h of aging (Figure S5).

An analysis of the bands of the malachite and azurite pigments in Figure 2B,C reveals that many of them show little change in intensity. In the region of 1380 cm^–1^, the ν_3_ CO_3_^2–^ ^34–36^ band of heat samples undergoes an increase in intensity when compared with the fresh paint, as shown in Figure 2. An increase in intensity is observed at 1490 and 1462 cm^–1^, which are related to ν_3_ CO_3_^2– ^,^34–36^ malachite and azurite, respectively (Figure 2). The increase in the intensity of the characteristic bands of the pigments can be explained by the curing process of the binder and the formation of cross-links, leading to a decrease in the intensity of the binder characteristic bands. In this way, most of the azurite and malachite pigments remain unchanged with accelerated aging. The results of the colorimetric analysis show that the Δ*E* of the malachite and azurite systems is considerably low, less than five units. Despite changes in the resin’s chemical environment, the pigments show little alteration in color after aging, which aligns with the preservation of most of their chemical structures.

The spectrum of copper acetate in Figure 2C is much more complex due to the large overlap between many bands characteristic of the binder and the pigment.^34,37,38^ A drastic change in the spectra is observed when comparing the fresh paint with the other thermal treatment samples. In Figure S2C, it is possible to identify the absence of the bands at 3466, 3368, and 3267 cm^–1^ attributed to the νOH of the pigment in the thermal treatment samples. In Figure 3, the bands at 1595 and 1419 cm^–1^ related to the ν_as_COO^–^ and ν_s_COO^–^ stretching of the pigment are also observed in the spectra of the thermally treated samples, but with much lower intensities, as well as δ CH at 1441 cm^–1^, where it is possible to observe the band with lower intensity in the thermally treated samples.

**Figure 3:**
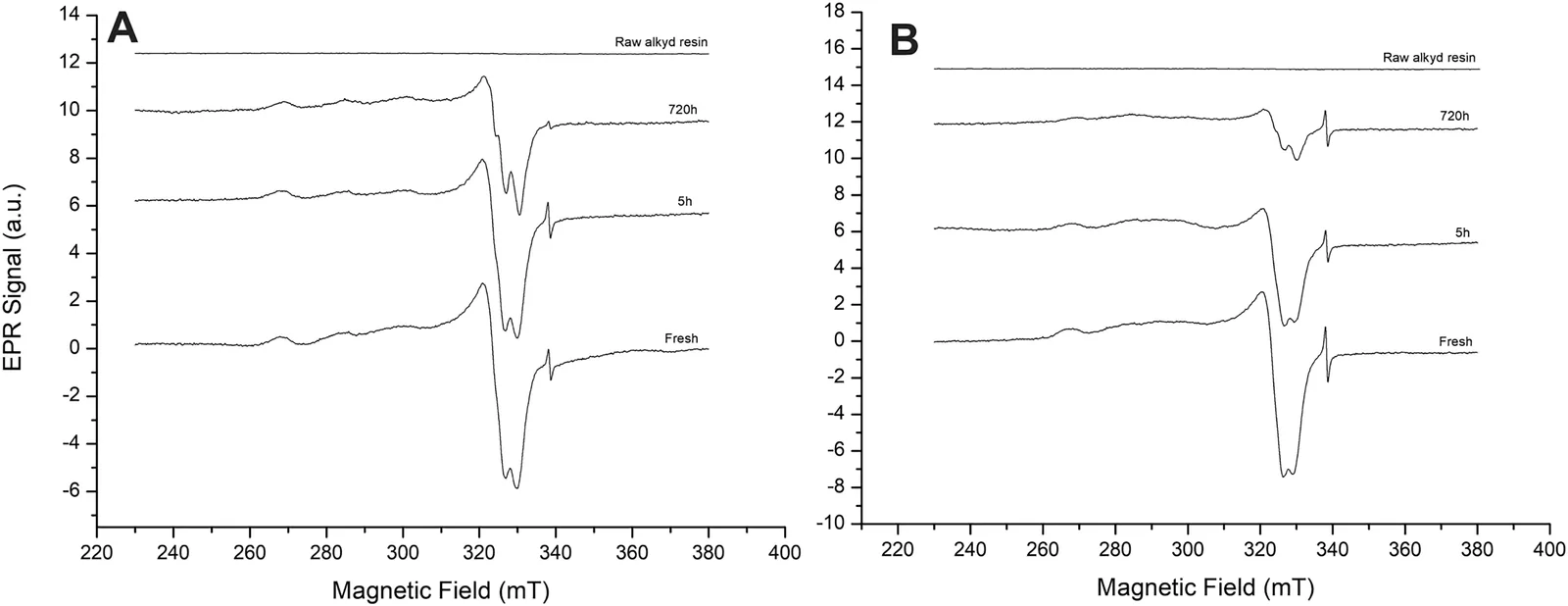
Experimental and simulated EPR spectra of the (A) malachite and (B) azurite systems in soybean oil alkyd resin.

Regarding the alkyd binder, a smaller difference in the decrease in band intensity was observed when compared to the azurite and malachite systems, as discussed below. The bands at 1722 and 1260 cm^–1^, attributed to the νC=O and νC–O–C aliphatic stretching of the ester, decreased in intensity after 360 h of aging, and the characteristic bands of the anhydride at 1579 and 1600 cm^–1^ also showed little variation in intensity as a function of time. The formation of a band at 1554 cm^–1^ can be attributed to the formation of copper soaps in the polymeric network,^36^ which was not observed in paints containing malachite and azurite. The formation of copper soaps in paints containing copper acetates was recently identified by Punis and co-workers in the linseed oil system.^39^

These drastic changes in the chemical environment of the pigment that are characterized by FTIR justify the greater colorimetric change of copper acetate compared to azurite and malachite, showing that soybean oil alkyd resin also changes the chromaticity of copper acetate, as shown in other studies with oily resins.^40,41^

Samples of the paints submitted under museum conditions were analyzed by FTIR for comparison with the accelerated aging samples. The spectra of the samples and the standards are present in Figure S6. Regarding the binder, its chemical structure was generally preserved when compared with the pure alkyd standard, and a small shift (3–4 cm^–1^) was observed in the ester carbonyl band, attributed to the early resin curing process. The presence of bands at 1600 and 1579 cm^–1^, attributed to the phthalic anhydride, and at 1120 and 1067 cm^–1^, attributed to the νC–H, νC–C, and νC–O stretching bonds of the alkyd resin (as discussed above), can be observed, showing that the resin backbone remains unchanged under these conditions.

All bands attributed to standard pigments (Figure S3) are observed in the paints submitted to museum conditions, demonstrating the preservation of their chemical structures under these conditions. These observations were expected for the malachite and azurite pigments, since even after thermal aging at 720 h, most of their chemical structure was preserved, as demonstrated by FTIR and μ-Raman analyses. For the copper acetate pigment, broad bands at 1595 and 1419 cm^–1^ related to ν_as_COO^–^ and ν_s_COO^–^ are observed, demonstrating that, unlike the thermal treatment samples, the museum conditions maintain the pigment’s structure for longer periods. Further details regarding the chemical environment of the copper centers present in the pigments are presented in Section 3.3.

To compare the influence of the pigment on the modification of the resin structure, the pure resin was subjected to thermal treatment for 720 h under the same conditions as the paints containing copper pigments. In the spectrum of the thermal treatment resin, Figure 2D shows the absence of the band attributed to the olefinic νC–H stretching at 3008 cm^–1^, the broadening and decrease in intensity of the bands at 2852 and 2922 cm^–1^ attributed to aliphatic νC–H bonds, the broadening and shift of the νC=O band from 1723 cm^–1^ in the fresh resin to 1730 cm^–1^ in the thermal treatment paint, and the decrease in intensity of the band at 1260 cm^–1^ attributed to aliphatic and aromatic moiety νC–O–C. These modifications can be attributed to the resin curing process, discussed previously, and observed in the spectra of the paints with copper pigments.

However, in the thermally treated resin, the presence of bands at 1600 and 1578 cm^–1^ attributed to the aromatic ring of phthalic anhydride and the δ C–H stretching outside the aromatic plane (740 and 704 cm^–1^) is noted with a low-intensity variation when compared with fresh resin, and paint samples with the presence of copper pigments, a decrease in the intensity of these bands is observed. Bartolozzi et al.^42^ demonstrate the same behavior in alkyd paint containing different inorganic pigments, and the same can also be observed in our work with copper pigments. This phenomenon is attributed to the progress of cross-link reactions during the aging of the alkyd resin and acts directly on the main chain containing phthalic anhydride.^42^ This process also occurs photochemically, as demonstrated by Pagnin et al.^8^

### 3.3 EPR Spectroscopy

The EPR technique is widely used to characterize the coordination environment of Cu­(II) centers in different fields due to its spin (nuclear (*I* = 3/2) and electronic (*s* = 1/2)) features.^43^ Recent advancements in the characterization of pigments in paints and cultural heritage using EPR have been noted in recent literature.^13,44,45^ In this work, the raw malachite and azurite pigments showed a broad signal (Figure S7). This isotropic signal results from large clusters containing Cu­(II) sites close to each other in high concentrations in the solid state and similar coordination bond distances.^45^ The similarity of the spectra is explained by the resemblance in the chemical structures of the two pigments, which only differ in their crystalline arrangement. In the malachite structure, the Cu­(II) centers occupy distorted octahedral sites, while in azurite, the Cu­(II) centers are arranged in two environments, one in a square planar and the other in a square-based pyramidal geometry.^46,47^ The same spectral pattern of the raw material was recently described by Hills-Kimball et al.^44^

When malachite and azurite pigments are mixed with soybean oil alkyd resin, changes in the spectrum are observed (Figure 3), and the presence of anisotropy was detected. In all spectra, three lines are resolved (*z*-component of the *g*-tensor) due to the hyperfine coupling resulting from the interaction of the unpaired electrons of the Cu­(II) center with the nuclear spin (*I* = 3/2) between 260 and 320 mT, and a strong line at 330 mT was attributed to the *x*- and *y*-components of the *g*-tensor. The spectral behavior with values in the region of *g*_//_ > *g*_⊥_> 2.0 indicates changes in the coordination environment of the copper centers. In both pigments, the metal–ligand bond undergoes axial elongation to form a geometry with the *D*_4*h*_ symmetry due to the Jahn–Teller distortion of the d^9^ center.^48–51^ A similar phenomenon was recently described by Hills-Kimball et al.^44^ with malachite and georgite pigments in the presence of linseed oil. In the spectra of the samples thermally treated for 720 h, it is possible to clearly observe the formation of a new signal at 325 mT and a broadening of the *z*-component of the *g*-tensor region, attributed to the formation of more than one copper species in the material, showing that part of the pigment in the fresh paint is maintained and a part has a change in its coordination environment.

Punis et al.^13^ and Hills-Kimball et al.^44^ presented the EPR spectra of the malachite and linseed oil system with the same characteristics found in the soybean oil alkyd resin. The authors attribute the change in the spectra of raw pigments (isotropic) and bound (axial) malachite to the possibility of the extraction of the Cu­(II) center by free carboxylates after the addition of the oily binder to the pigment without aging. Hills-Kimball et al.^44^ present the formation of a band at 1568 cm^–1^ after mixing and attribute the interactions to the formation of Cu­(II)-carboxylate complexes. Izzo et al.^52^ show that the formation of copper carboxylates can be identified by the stretching ν_as_COO^–^ between 1588 and 1584 cm^–1^ and ν_s_COO^–^ 1417 and 1421 cm^–1^. However, in our study, the formation of free or complexed carboxylates with the Cu­(II) center generated by the soybean oil alkyd resin was not clearly identified for the malachite and azurite pigments. However, the interaction with the −O groups donors with the Cu­(II) center can occur through other oxygenated species formed during the curing process of the alkyd resin, in addition to secondary and volatile species being generated, such as ketones, aldehydes, etc., which can act as ligands for the copper centers in the mixture.^53^ It is interesting to highlight that even with these interactions identified by EPR, there is no significant chromatic modification in the paints containing malachite and azurite, even in the thermally treated samples, as demonstrated in the colorimetric tests.

Figure 4 shows the experimental EPR spectra for the copper acetate systems that were raw, fresh, and thermally treated for 5 and 720 h.

**Figure 4:**
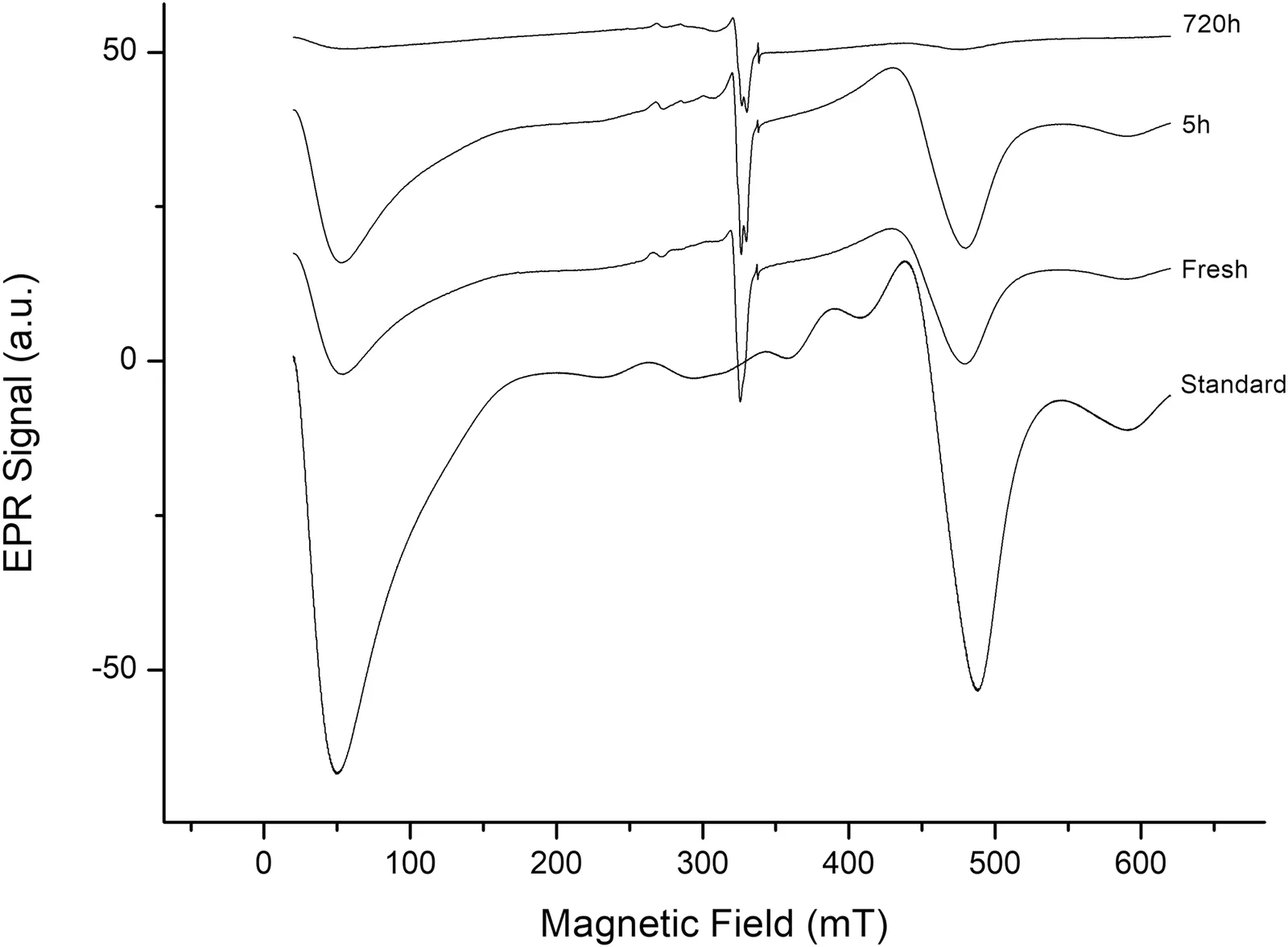
Experimental EPR spectra of the copper acetate from 0 to 700 mT of the standard, fresh, and thermally treated for 5 and 720 h.

The spectra of the copper acetate/alkydy resin system show significant changes. It can be observed that the spectrum of the standard copper acetate pigment (Figure 4) presents three characteristic signals: 600, 500 mT, and a broad signal between 0 and 100 mT; the latter was attributed to a forbidden transition and characterized the dinuclear nature of the compound. This spectral pattern confirms that the raw pigment is in the dimeric form, as previously reported in the literature,^14,54^ with the two Cu­(II) centers in *D*_4_ *_h_* symmetry bridged by four acetate groups.^55^

In the presence of soybean oil alkyd resin, the spectrum presents new signals in the region between 260 and 340 mT (Figure 4), which can be attributed to the formation of monomeric species. This is further supported by the disappearance of the forbidden transition associated with the dimeric form. It is possible to note the presence of three signals in the *g*_//_ region and two intense signals in the *g*_⊥_ region, typical of monomeric Cu­(II) species. The transition from the dimeric to the monomeric form of copper acetate is reported for several binders such as oils, gums, proteins,^54,39,14,12,41^ and now in alkyd resin.

Values of *g*_//_ > *g*_⊥_ > 2.0 are observed, corroborating an elongation along the *z*-axis due to the Jahn–Teller distortion of the d^9^ center, similar to the spectra of fresh and malachite and azurite with thermal treatment.^48–50^ Unlike malachite and azurite, in the system with copper acetate, the formation of a broad band at 1554 cm^–1^ was identified in FTIR and can be attributed to the formation of copper soaps and contributed to the formation of monomeric species observed in EPR spectra. It is noted that a tendency toward a more rhombic spectrum was identified in the sample thermally treated for 720 h containing copper acetate.

Another important observation is that with the passage of aging time, the EPR signals associated with the dimeric species of copper acetate decrease and those associated with the monomeric species increase. Recent studies involving copper acetate and linseed oil^39^ have shown that the process of monomer formation involves the dissolution of dimeric species within the binder medium. This is followed by the breakdown of these species by molecules containing donor atoms, such as fatty acids. A similar behavior has been observed here in our study using soybean oil alkyd resins. Thus, in the presence of soybean oil alkyd resin, copper acetate proves to be less chemically stable when compared with malachite and azurite, as well as in the presence of other binders described previously, which reflects the large chromatic differences when compared to malachite and azurite (Δ*E* 2.77 and 3.72, respectively), while in acetate it has a Δ*E* equal to 11.19, drastically varying the color from blue-green to greenish yellow.

The EPR spectra of the paintings submitted to museum conditions can be seen in Figure S9. In the painting containing malachite, the lines attributed to hyperfine coupling of the Cu­(II) center can be observed, similar to the fresh system shown in Figure 3A. The spectrum of the painting containing azurite presents a signal at 290 mT and an intense signal at 330 mT, which can be attributed to interactions between the paramagnetic sites. In both paintings, an axial spectrum with *g*_//_ > *g*_⊥_> 2.0 is observed, corroborating the spectra obtained for the thermally treated samples.

In the spectrum of paint containing copper acetate under museum conditions, it is possible to observe signals at 600 mT and a broad signal between 0 and 100 mT, attributed to the copper dimer (discussed previously). However, it is also possible to observe the presence of three signals in the *g*_//_ region and two intense signals in the *g*_⊥_ region, typical of monomeric Cu­(II) species. However, when compared with the sample thermally treated for 720 h, it is noted that the signals related to the dimeric species have a greater intensity, demonstrating that temperature and time affect the conversion of the dinuclear unit to the copper monomer in these paints.

### 3.4 Scanning Electron Microscopy

The SEM-EDS technique is widely used to characterize materials constituting cultural heritage,^56,57^ including pictorial layers in ancient and contemporary paintings containing pigments of various colors, including greens such as malachite,^58–60^ and it is also possible to identify aggregates attributed to the binder–pigment interaction.^61^ Figure 5 shows micrographs of the paint samples containing soybean oil alkyd resin with the pigments of malachite, azurite, and copper acetate.

**Figure 5:**
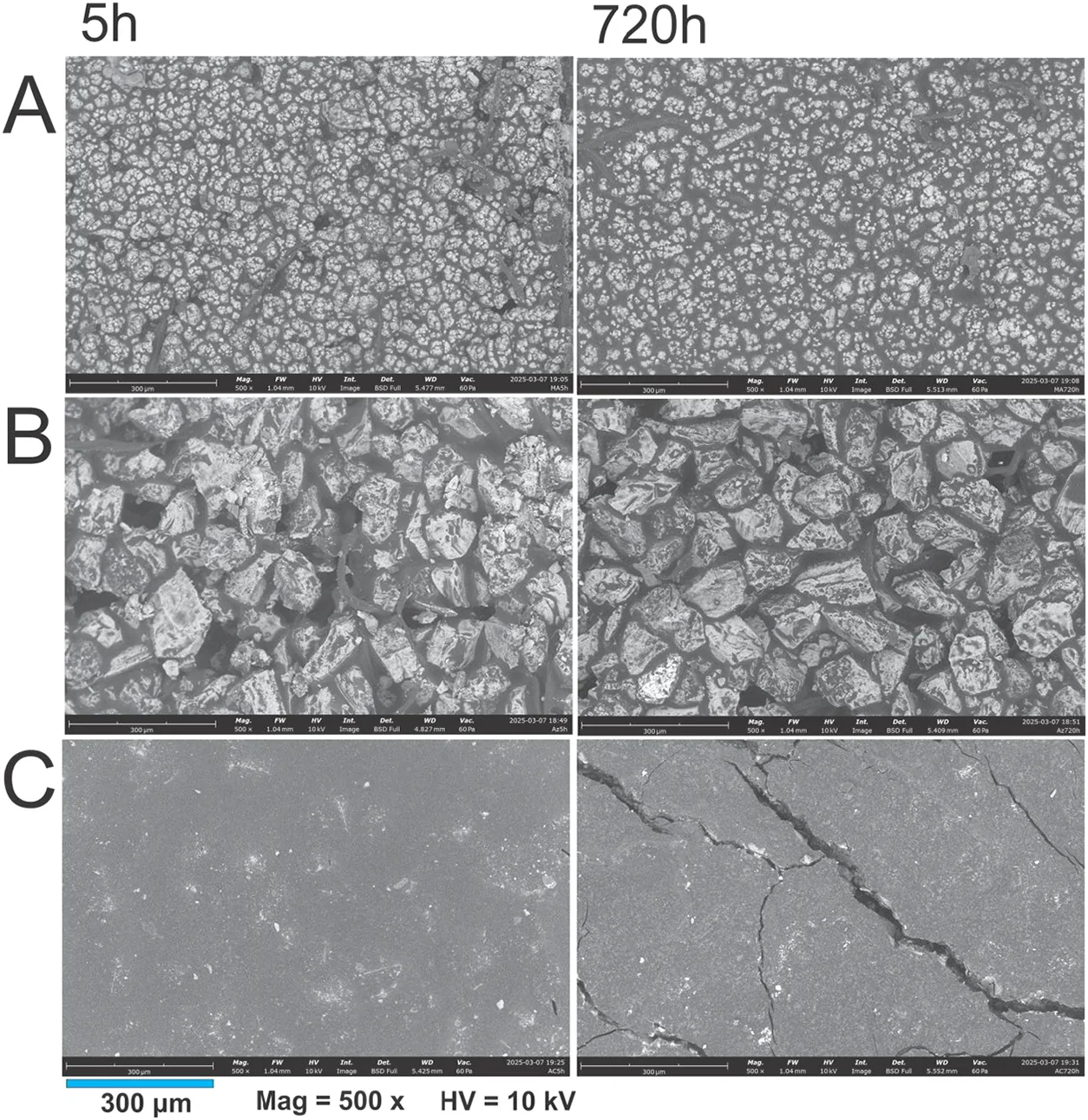
Micrographs of (A) malachite, (B) azurite, and (C) copper acetate samples in soybean oil alkyd resin thermally treated for 5 and 720 h.

A uniform particle-size distribution is observed with few variations at the beginning and end of aging. Figure S10 (Supporting Information) shows micrographs of malachite and azurite samples in soybean oil alkyd resin thermally treated for 5 and 720 h, with particle-size distribution histogram and the presence of pigments in the paint layers highlighted in red. The samples containing malachite exhibit a lateral particle-size distribution of 27.21 μm ± 6.11 and 25.40 μm ± 3.94 for the samples thermally treated for 5 and 720 h, respectively. In the samples containing azurite, the lateral size identified was 62.97 μm ± 15.04 and 75.84 μm ± 14.66 for the samples thermally treated for 5 and 720 h, respectively. The quantitative distribution results show that there are no significant changes in the lateral particle size at the beginning or end of the aging of paint layers. In both samples, there is no evidence of the formation of agglomerates, with a significant increase in size attributed to the interaction between binder and pigments. This observation is in accordance with the spectroscopic data. Such findings confirm that the alkyd resin provides a stable physical matrix that preserves pigment particle integrity under thermal stress, aligning with the minimal chemical changes detected by FTIR and EPR spectroscopy for malachite and azurite.

In contrast, the paint layers containing copper­(II) acetate exhibited a distinct morphology and exhibited a uniform layer. Although pigment particles were not clearly discernible at the magnifications employed, the samples thermally treated for 720 h displayed noticeable cracking and microfissures within the resin layer.

These morphological alterations likely arise from the chemical instability of copper acetate in the resin, promoting mechanical stress due to volumetric changes and the generation of brittle degradation products. This observation supports the significant colorimetric (Δ*E* > 11) and spectroscopic changes discussed previously.

### 3.5 Raman Spectroscopy

Raman spectroscopy was employed to investigate the molecular structures present in the fresh and thermally treated paint systems. The collected spectra for malachite, azurite, and copper­(II) acetate pigments, as well as the respective paints thermally treated for 5 and 720 h, are shown in Figure 6, and the Raman spectrum of pure soybean oil-based alkyd resin is provided in Figure S11 the Supporting Information.

**Figure 6:**
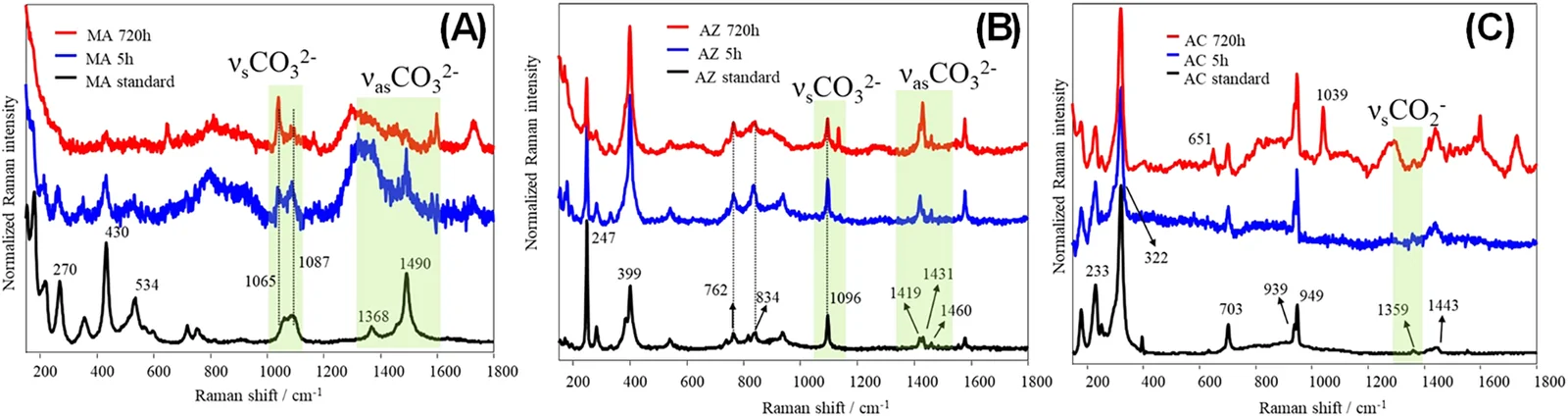
Raman spectra of (A) malachite, (B) azurite, and (C) copper acetate standards and the paints containing each pigment in soybean oil alkyd resin thermally treated for 5 and 720 h, with the laser line at 532 nm.

Basically, the spectra can be divided into two regions: low frequencies (below ∼600 cm^–1^) are assigned to lattice modes, while higher frequencies (above ∼600 cm^–1^) are assigned to internal modes. The standard malachite pigment exhibited strong and characteristic Raman signals at 270 cm^–1^ (O–Cu–OH bending), 430, and 534 cm^–1^ (Cu–O stretching mode). In addition, an intense band is observed at 1494 cm^–1^ and two less intense bands at 1368 and 1461 cm^–1^ are assigned to CO_3_^2–^ antisymmetric stretching modes. In this region, four bands can be expected; however, in our case, only three were observed. Complementarily, the CO_3_^2–^ symmetric modes are observed at 1065 and 1087 cm^–1^.^62–64^

Azurite presents the CO_3_^2–^ asymmetric stretching modes at 1419, 1431, and 1460 cm^–1^ and CO_3_^2–^ symmetric stretching mode at 1096 cm^–1^ in accordance to the literature.^64^ Also, the Raman spectrum shows typical bands at 247, 399, 762, and 834 associated with its layered carbonate structure.^65^

Copper­(II) acetate showed dominant bands at 233 and 322 cm^–1^ assigned to lattice modes and other bands at 703 cm^–1^ related to (CO_2_^–^ bending) and 949 cm^–1^ attributed to C–C stretching, which split into two components (949 and 939 cm^–1^), as observed for cobalt acetate.^65,66^ The CO_2_^2–^ symmetric stretching is seen at 1359 cm^–1^ and increases with aging; the CH_3_ bending is seen at 1443 cm^–1^.

In the paints containing malachite and azurite, the Raman spectra collected after 5 and 720 h of aging retained the principal bands of the original pigments, indicating the preservation of their chemical structures within the alkyd matrix. Notably, the signal-to-noise ratio decreases considerably due to external influences like soybean oil-based alkyd resin and linen canvases. Nevertheless, the region between 1050 and 1100 cm^–1^ is split into two bands at 1041 and 1089 cm^–1^ for both malachite samples (5 and 720 h). Such a region is related to CO_3_^2–^ and can be attributed to different environments. In the case of azurite samples (5 and 720 h), one can observe the inversion in the intensity of the bands at 247 and 399 cm^–1^ with respect to the standard. These low-frequency signals are related to lattice modes, and we may attribute them to Cu–O vibrational modes of new copper-based coordination species formed during the resin curing and aging processes. Such transformations are consistent with the EPR observations of changes in the copper­(II) coordination environment and confirm limited interaction without major degradation of the pigments themselves.^39^ In addition, the stretching modes of carbonate paints were affected upon aging. In the case of azurite, in general, all of the bands are broadened with aging, although the degradation can be discarded.

In the copper­(II) acetate system, more pronounced changes were detected. After 720 h of aging, a new prominent band emerged at 651 cm^–1^, which is assigned to CO_2_^–^ deformations of newly formed copper-carboxylate complexes, in agreement with FTIR results.^66,67^ In addition, a medium-intensity band emerged at 1039 cm^–1^, assigned to −CH_3_ deformation, although it is expected to be less intense at this vibration.

Moreover, in the samples of azurite and copper­(II) acetate after aging, slight changes at low wavenumbers can be observed, as shown in Figure S12. For instance, azurite samples showed a shoulder at 236 cm^–1^ and a band at 264 cm^–1^, in addition to a shoulder at 213 cm^–1^ in copper­(II) acetate. These bands are related to the lattice modes, and we can speculate that such changes are due to the Cu–O bonds.

The Raman data thus corroborate the spectroscopic (FTIR, EPR) and colorimetric findings, demonstrating that while malachite and azurite exhibit strong structural resilience within the soybean alkyd system. The incorporation of a copper acetate pigment or the formation of copper soaps leads to structural changes and chromatic alterations.

## 4 Conclusion

In this work, the experiments shown that malachite and azurite have the chemical stability to be employed as a pigment in the presence of a soybean oil-based alkyd resin. On the other hand, copper acetate undergoes structural modification, changing from a dinuclear to a mononuclear species in the presence of the resin, resulting in the formation of soap and making it unsuitable for use in paints. Despite changes in the resin’s chemical environment, the layers containing malachite and azurite exhibited minimal color change after aging, from both molecular and macroscopic points of view, different from the observed in studies using phthalo blue (PB15) and phthalo green (PG7) in the presence of alkyd resin, preserving most of their chemical structures. The results suggest that malachite and azurite have structural resilience in soybean oil-based alkyd resin that can be a viable and cost-effective alternative for use as a binder in paint new formulation with these pigments.

## Supplementary Material



## Notes

Colorimetric table values, FTIR and EPR of standard materials used in this work, FTIR of the samples in the 1900–1600 and 770–690 cm^–1^ ranges, FTIR and EPR of paints submitted at museology conditions, and Raman spectra in the 100–350 cm^–1^ range (PDF)
