# Supplementary material for: Determination of Molecular Interactions between Copper Pigments and Soybean Oil Alkyd Resins in Paints: A Spectroscopic Study
Source: ACS Omega. 2026 May 7;11(19):27751–61. doi: 10.1021/acsomega.5c05576 (PMC13557265; doi:10.1021/acsomega.5c05576)
Supplement: Supplementary file 1 [file ao5c05576_si_001.pdf]

Determination of molecular interactions between copper pigments and soybean oil alkyd resins in paints: a spectroscopic study

Thiago Guimarães Costa*[a,b], António José Estêvão Grande Candeias[b], Enzo Gimenes Gazotto[a], Feik Amil de Campos Júnior[d], Leonardo Negri Furini[d], Adolfo Horn Jr.*[c]

[a] Laboratory of Materials, Atelier for Conservation and Restoration of Cultural Heritage, Santa Catarina State Culture Foundation, 88025-200 Florianópolis – SC, Brasil

[b]HERCULES Laboratory, Institute for Advanced Studies and Research, University of Évora, Largo Marquês de Marialva 8, Évora 7000-809,Portugal

[c] Department of Chemistry, Federal University of Santa Catarina (UFSC), 88040-900 Florianópolis, SC, Brasil

[d] Department of Physic, Federal University of Santa Catarina (UFSC), 88040-900 Florianópolis, SC, Brasil

----

Supporting Information

Table S1. Values of L, a, b and ΔE for paints containing soy alkyd resin and aged malachite, azurite and copper acetate pigments

Time aged (hours)	L*	a*	b*	ΔE
**Copper acetate**				
Fresh sample	7.6	-4.5	6.3	0
5	12.1	-4.1	-9	15.95
24	12.9	-4.9	-10	1.51
168	13.3	-8.1	-8.4	3.60
360	16.3	-6.1	-4.7	5.17
720	17.4	-5.5	1	5.84

Δ overall (720h - fresh)	9.8	-1	-5.3	11.19
**Azurite**				
Fresh sample	5.1	12.4	-26.2	0
5	5.6	19.4	-32.6	9.50
24	5.1	19.9	-33.6	1.23
168	12.5	22.2	-38.6	9.22
360	6.1	16.2	-30.1	12.21
720	6.4	15.5	-27.8	2.42
Δ overall (720h - fresh)	1.3	3.1	-1.6	3.72
**Malachite**				
Fresh Sample	51.8	-33.5	7.3	0
5	51.1	-35.6	3.6	4.31
24	51.6	-34.3	3.6	1.39
168	54.1	-36.0	3.1	3.06
360	54.7	-35.3	3.1	0.92
720	49.4	-34.2	8.5	7.65
Δ overall (720h - fresh)	-2.4	-0.7	1.2	2.77

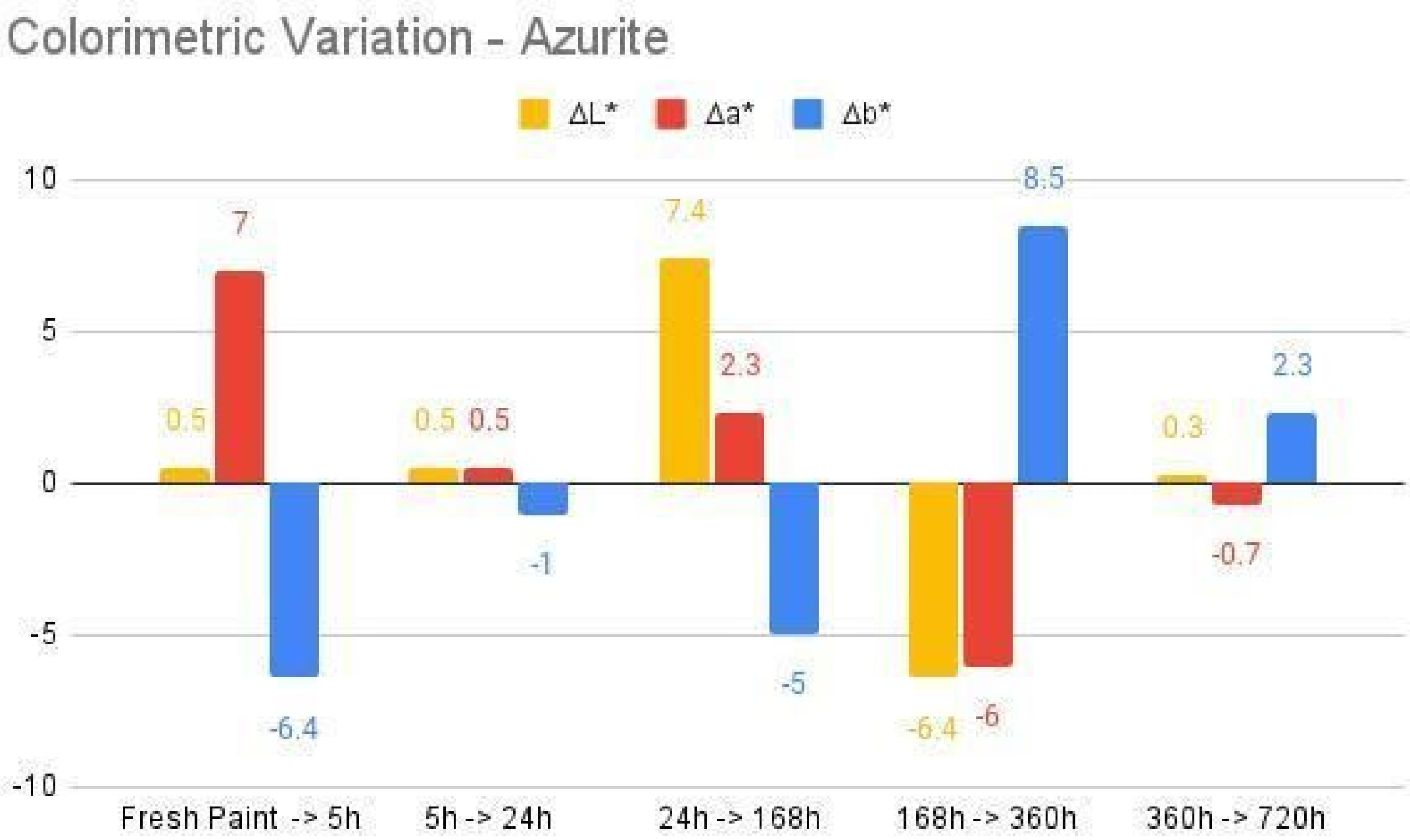
Colorimetric Variation - Azurite
ΔL*
Δa*
Δb*
10
5
0
-5
-10
7
7.4
8.5
0.5
0.5
0.5
2.3
2.3
0.3
-1
-0.7
-5
-6.4
-6.4
-6
Fresh Paint -> 5h
5h -> 24h
24h -> 168h
168h -> 360h
360h -> 720h

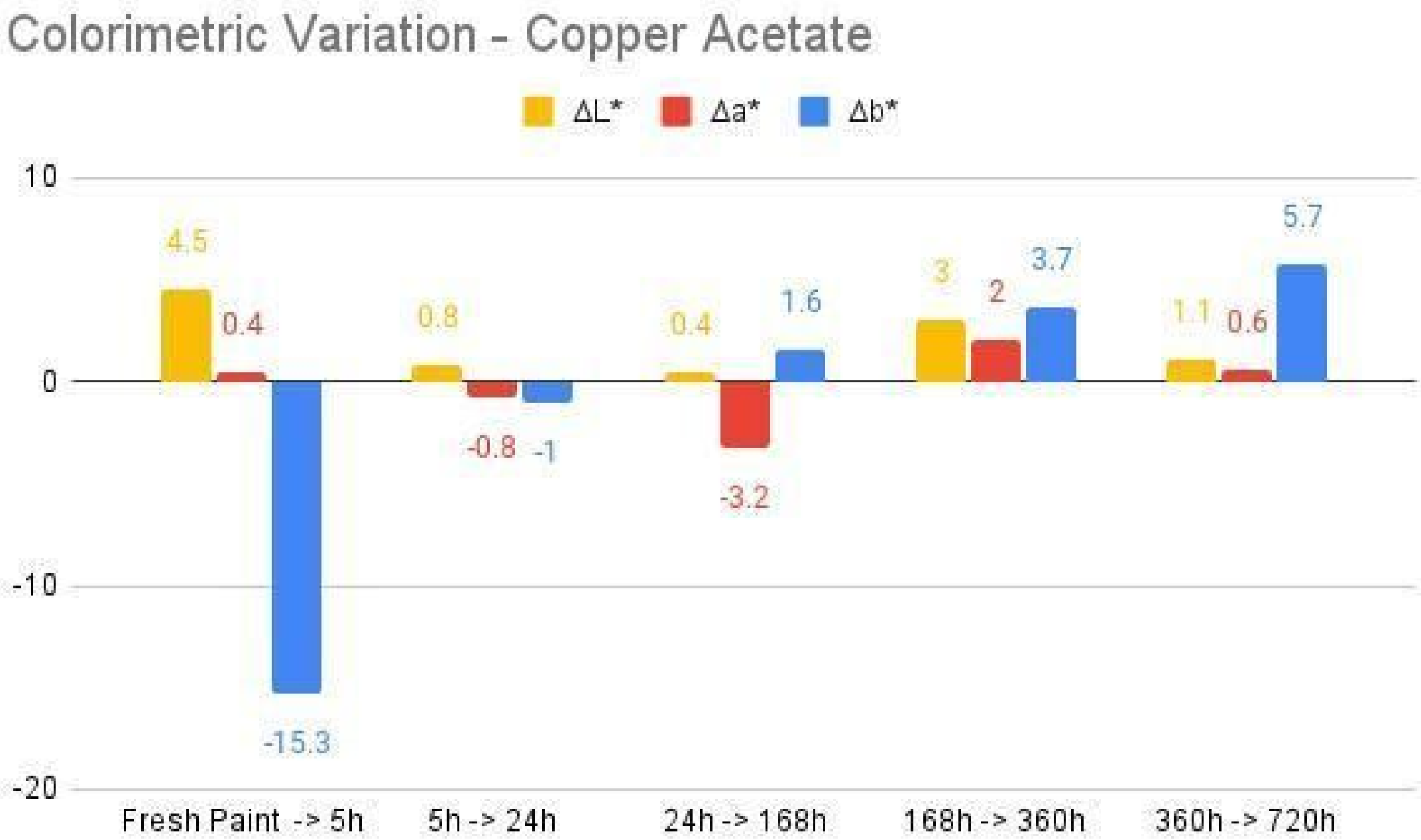
Colorimetric Variation - Copper Acetate
ΔL*
Δa*
Δb*
10
0
-10
-20
4.5
0.4
0.8
0.4
1.6
3
2
3.7
1.1
0.6
5.7
-0.8
-1
-3.2
-15.3
Fresh Paint -> 5h
5h -> 24h
24h -> 168h
168h -> 360h
360h -> 720h

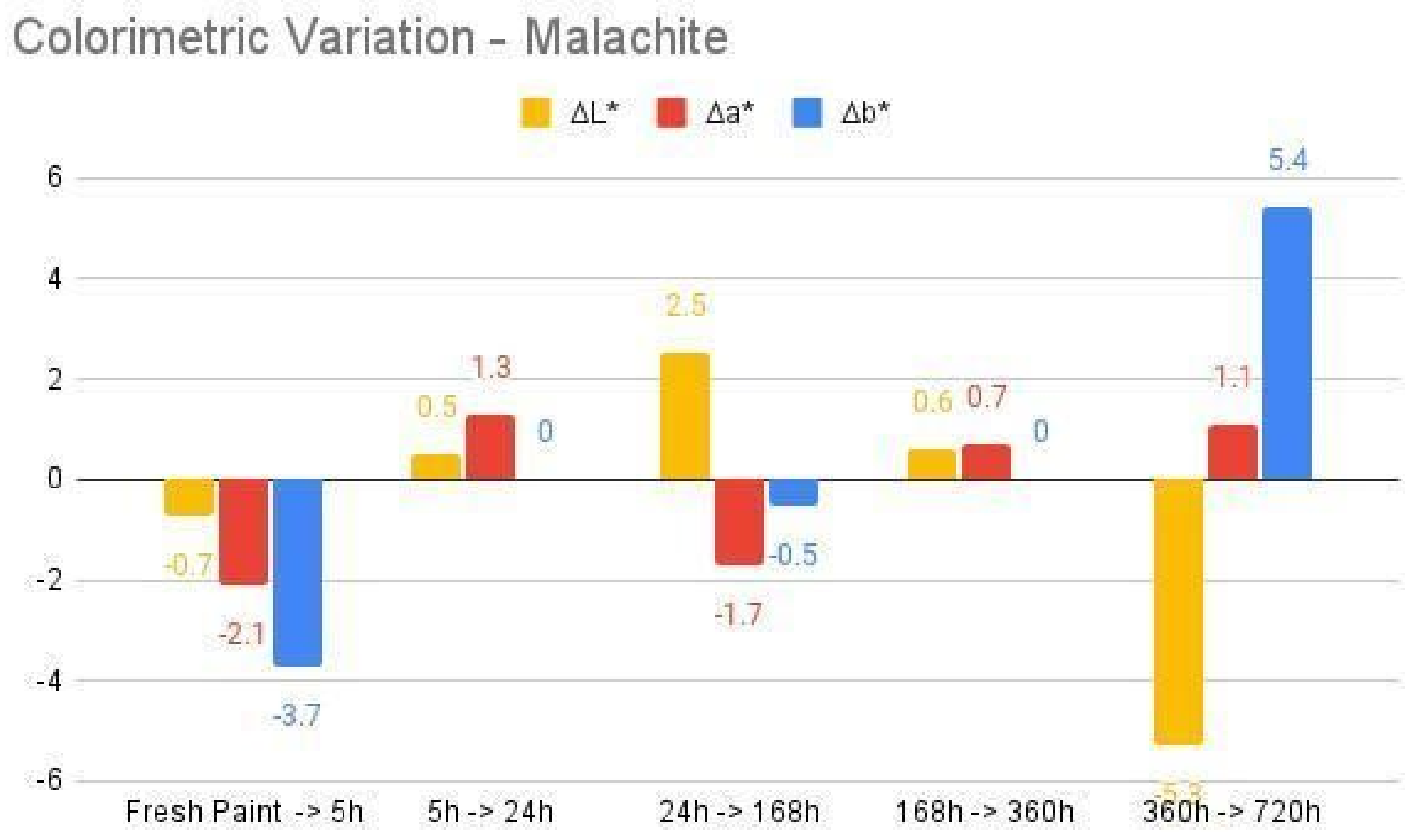

Figure S1. Variation of a*, b*, L* at each point for alkyd paints containing copper pigments

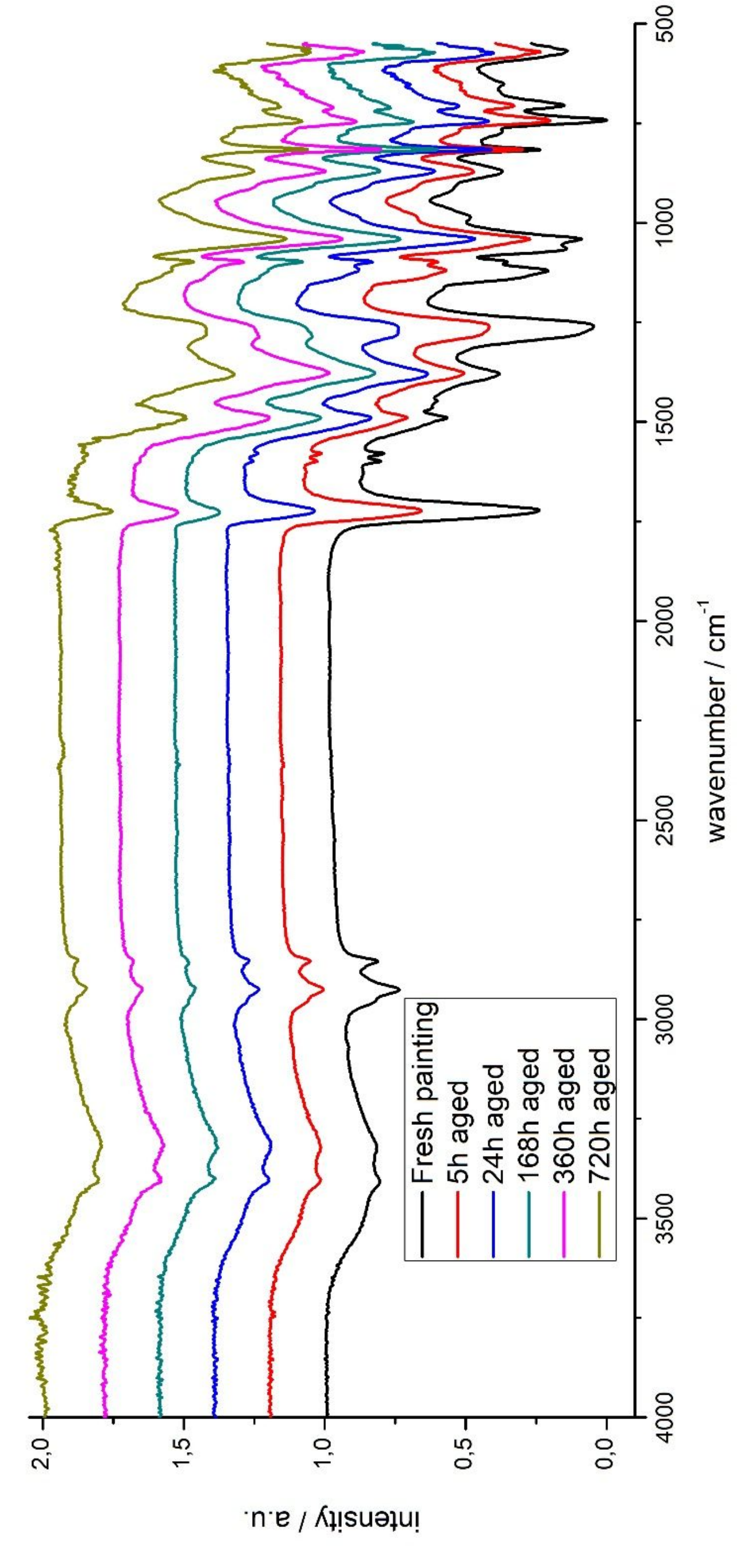

Figure S2-A: FTIR spectra of aged soybean resin alkyd paints containing malachite pigment.

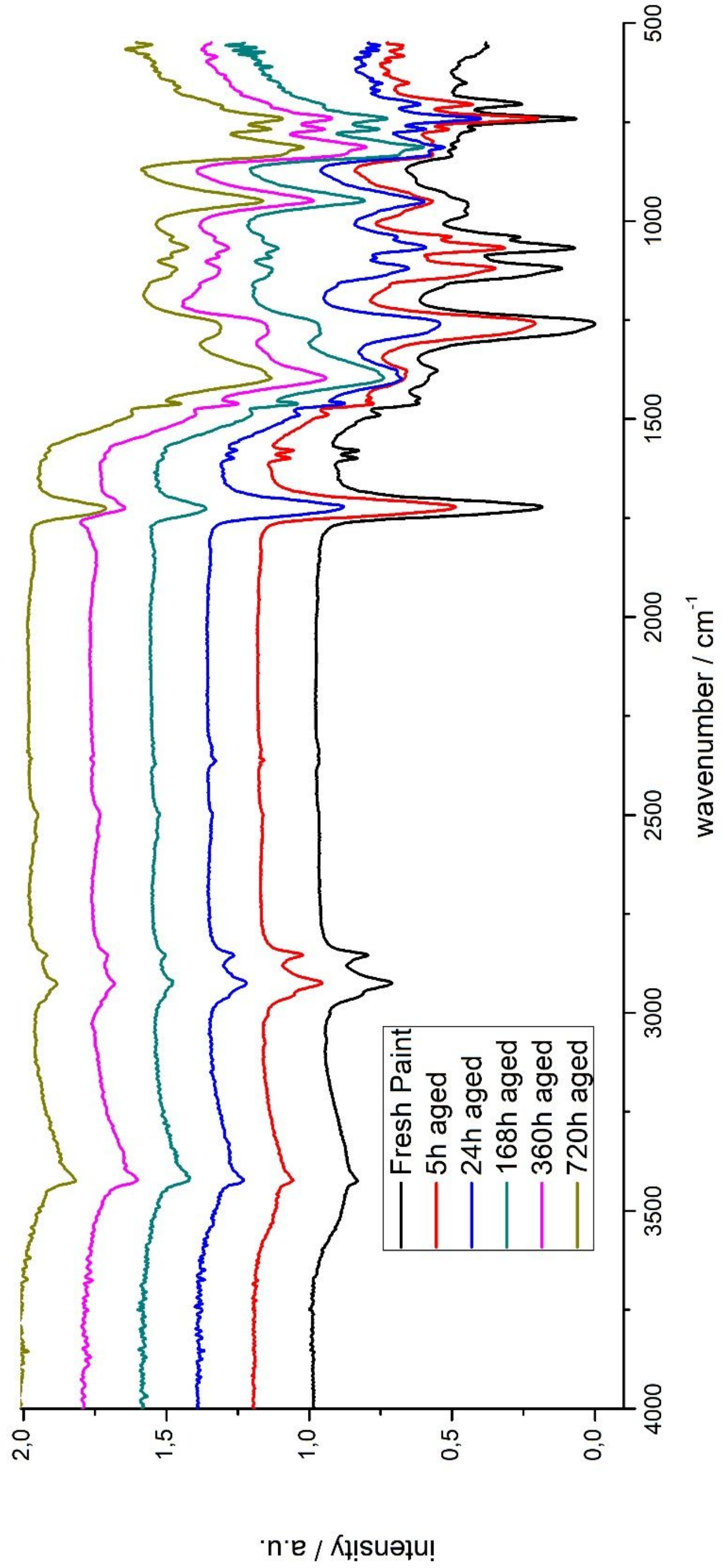

Figure S2-B: FTIR spectra of aged soybean resin alkyd paints containing azurite pigment.

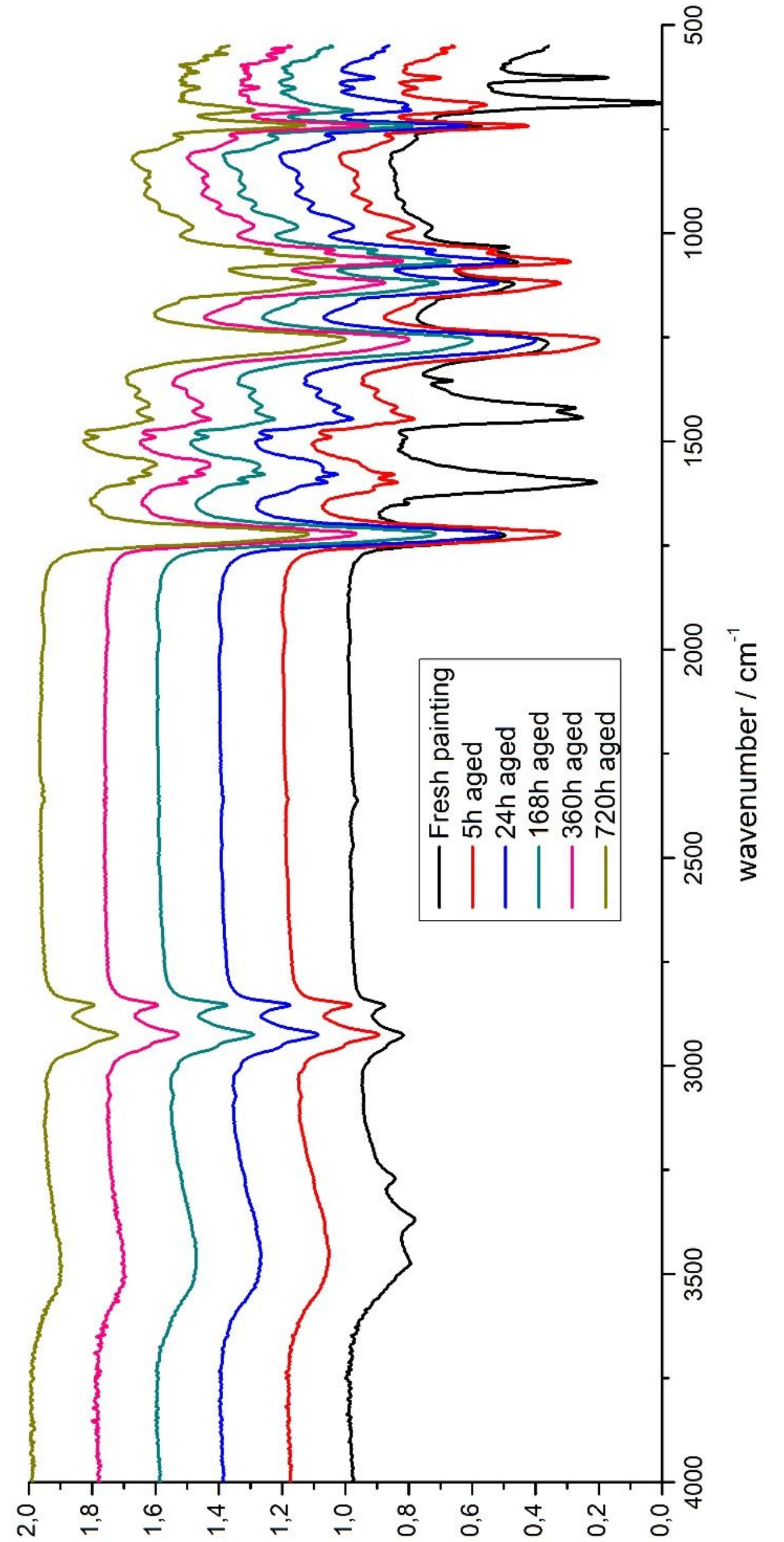

Figure S2-C: FTIR spectra of aged soybean resin alkyd paints containing copper acetate pigment.

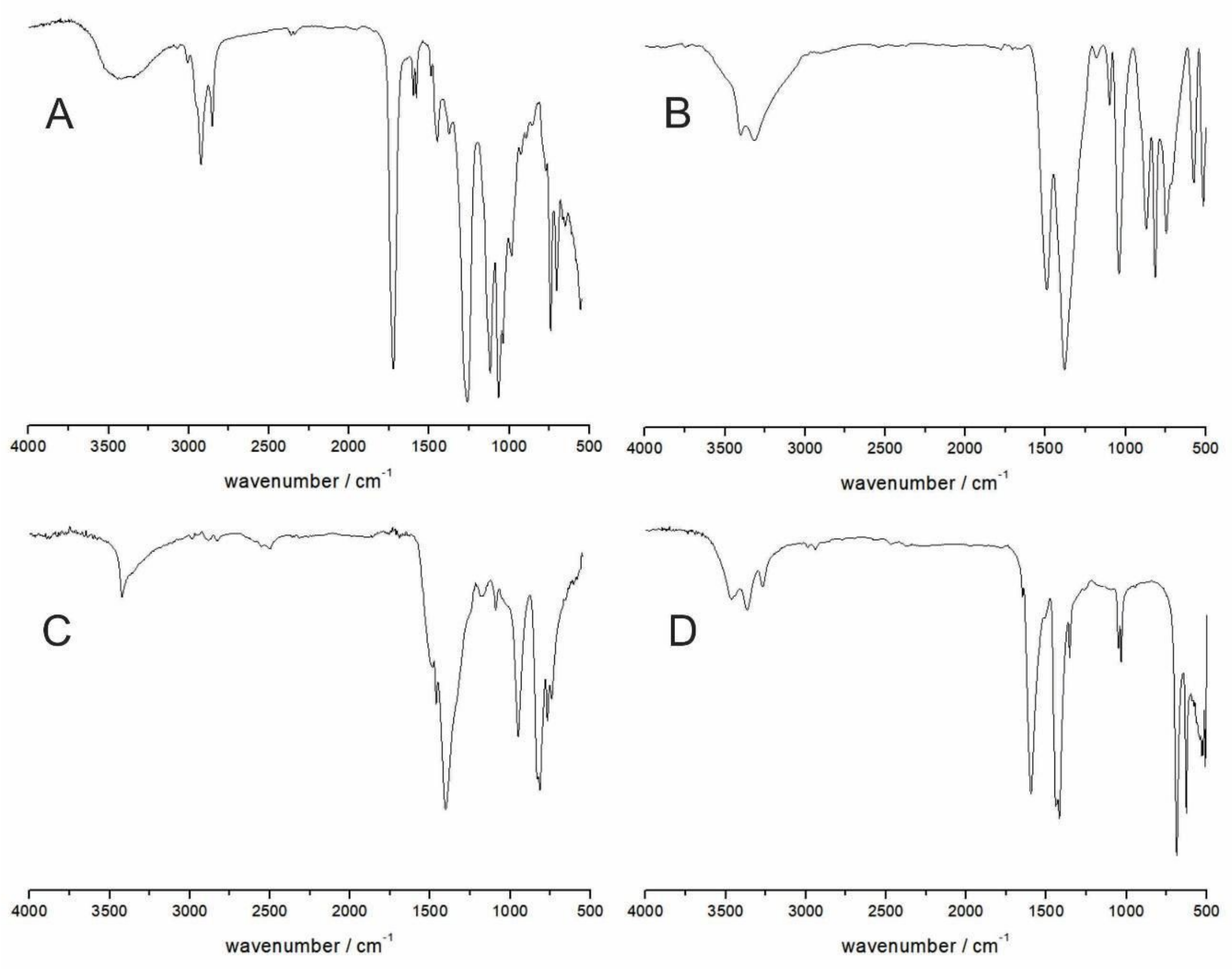

Figure S3. FTIR spectra of the pure materials used in this work, where A = soybean oil alkyd resin; B = malachite; C = azurite; D = copper acetate

Table S2. Assignment of the frequencies found in standard materials used in this work and according with ref. 1, 2 and 3.

Frequency (cm^{-1})	Assignment
**Soybean Oil Alkyd Resin**	
3180-3550	ν O-H
3008	Olefinic ν C-H
2922	Aliphatic ν C-H
2852	Aliphatic ν C-H
1723	ν C=O frequency of ester
1600	ν C=C alkene an aromatic band
1579	ν C=C alkene an aromatic band
1487	δ C-H methyl groups
1376	δ C-H methyl groups
1260	ν C-O-C aliphatic and aromatic moiety
1040	ν C-O-C aliphatic and aromatic moiety
740	Out of plane aromatic δ C-H
704	Out of plane aromatic δ C-H
**Azurite**	
1093	ν_1 CO_3^{2-}
1402	ν_3 CO_3^{2-}
1462	ν_3 CO_3^{2-}
3422	OH
**Malachite**	
1041	δ OH
1097	ν_1 CO_3^{2-}
1377	ν_3 CO_3^{2-}
1490	ν_3 CO_3^{2-}

3317	OH
3400	OH
**Copper Acetate**	
1031	ν CH
1051	δ CH
1353	δ CH
1419	ν_s COO
1444	δ CH
1595	ν_{as} COO
3267	ν OH
3368	ν OH
3466	ν OH

[1] Buti, David & Rosi, Francesca &Brunetti, Bruno &Miliani, Costanza. (2013). In-situ identification of copper-based green pigments on paintings and manuscripts by reflection FTIR. Analytical and bioanalytical chemistry. 405. 10.1007/s00216-013-6707-6.

[2] Mohamed E. ELBA, Elsayed M. ABDEL REHİM, Ramadan E. ASHERY*, Synthesis and characterization of alkyd resin based on soybean oil and glycerin using zirconium octoate as catalyst, Damanhour University, Faculty of Science, Department of Organic Chemistry, 2018, International Journal of Chemistry and Technology, http://dergipark.gov.tr/ijct

[3] Sibokoza, Simon Bonginkosi&Moloto, M.J. &Mtunzi, Fanyana&Moloto, Nosipho. (2021). Thermal Decomposition of Copper Acetate at Various Temperature and Time to form Copper Oxide/Copper Nanoparticles. Asian Journal of Chemistry. 34. 239-244. 10.14233/ajchem.2022.23495.

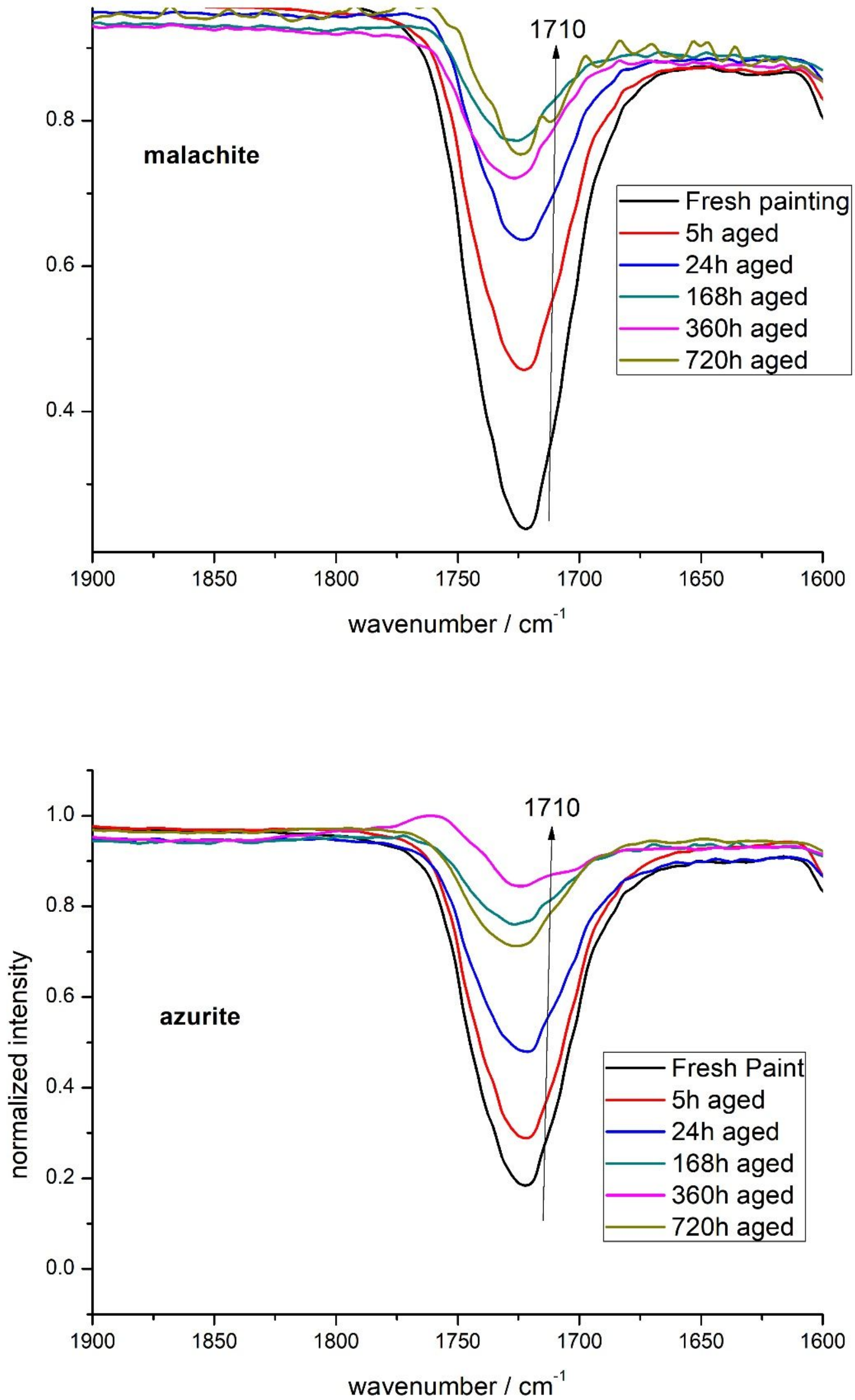

Figure S4. FTIR spectra of malachite and azurite in presence of alkyd resin at different age times in the 1900 - 1600 cm^{-1} range.

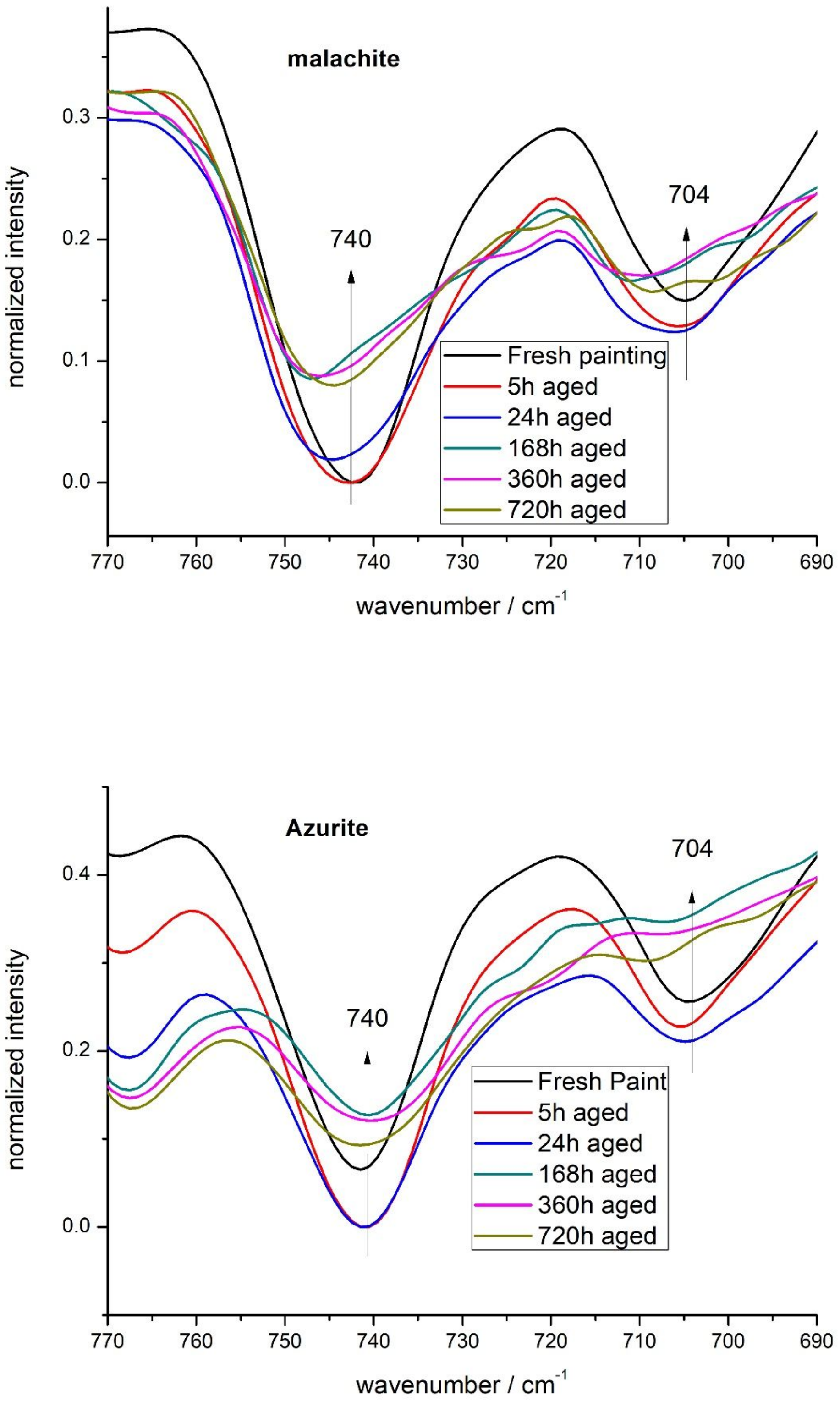

Figure S5. FTIR spectra of malachite and azurite in presence of alkyd resin at different age times in the 770 – 690 cm^{-1} range.

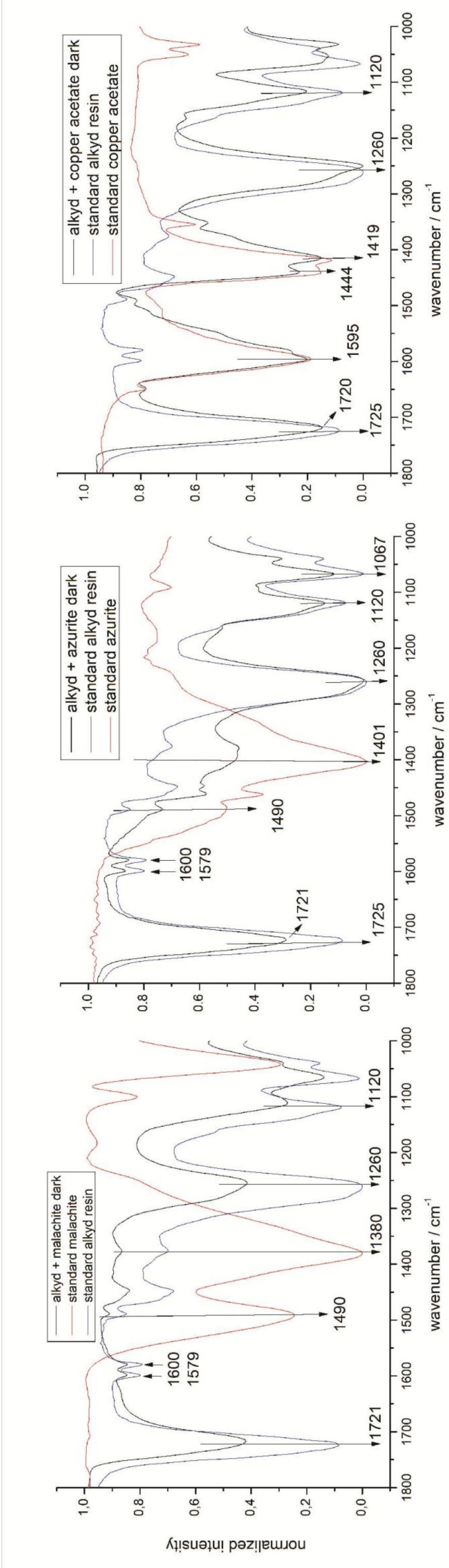

Figure S6. FTIR of paints submitted at museology conditions (25 ± 3 °C and 55% ± 5%) at minimum 720h and comparation with the pigment and binder resin standards.

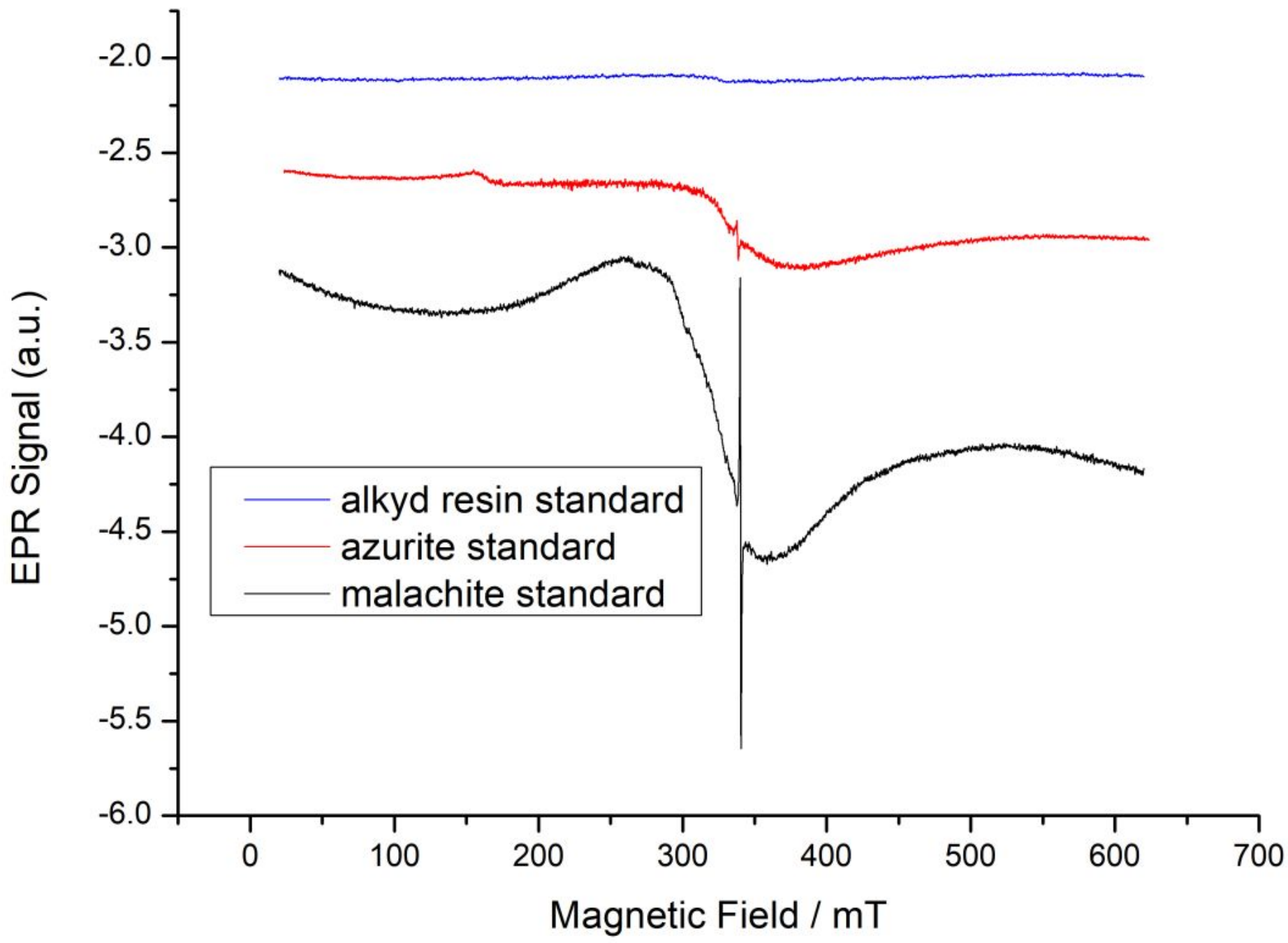

Figure S7. EPR spectra of malachite, azurite and alkyd resin standards.

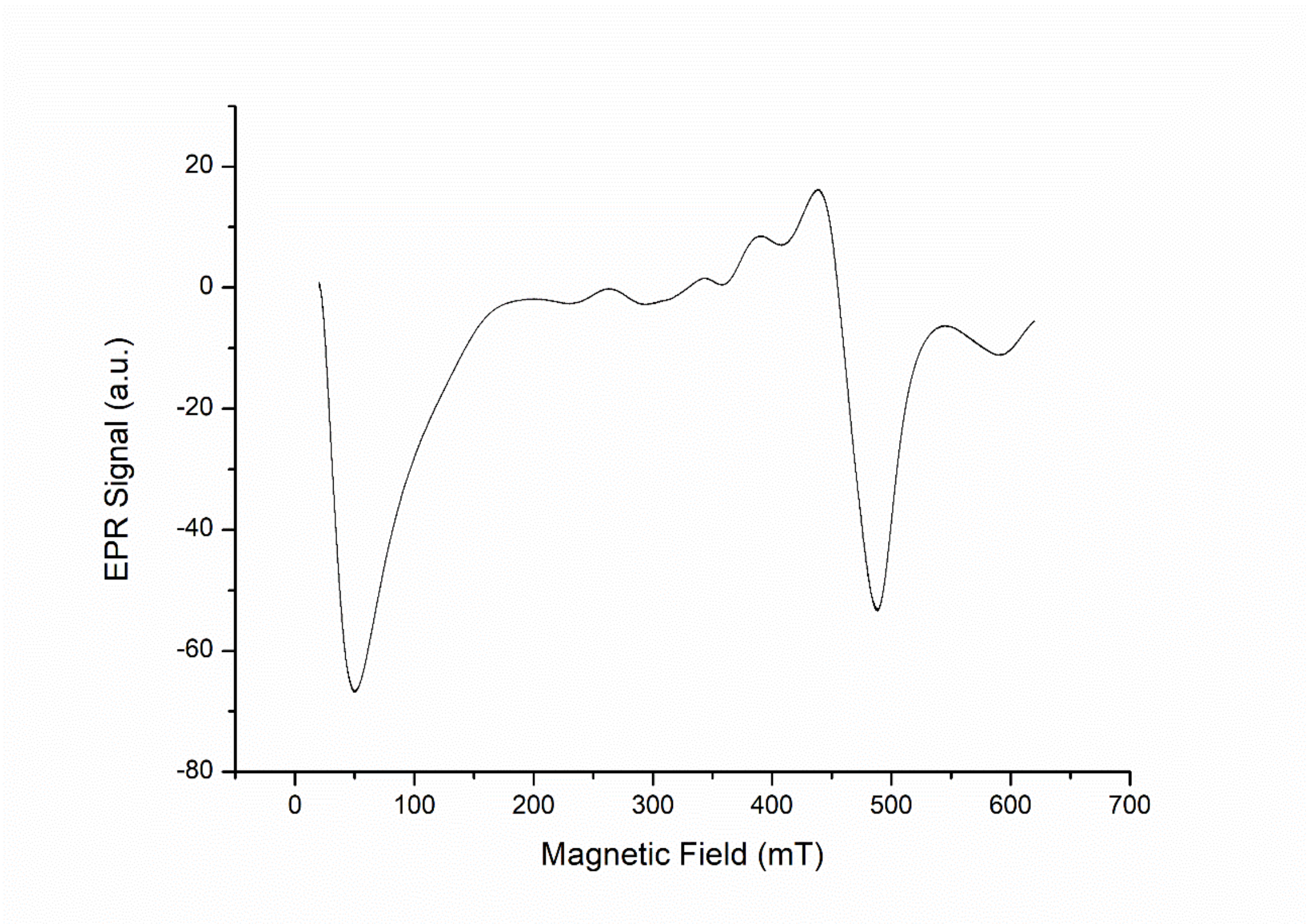

Figure S8. EPR spectra of raw copper acetate

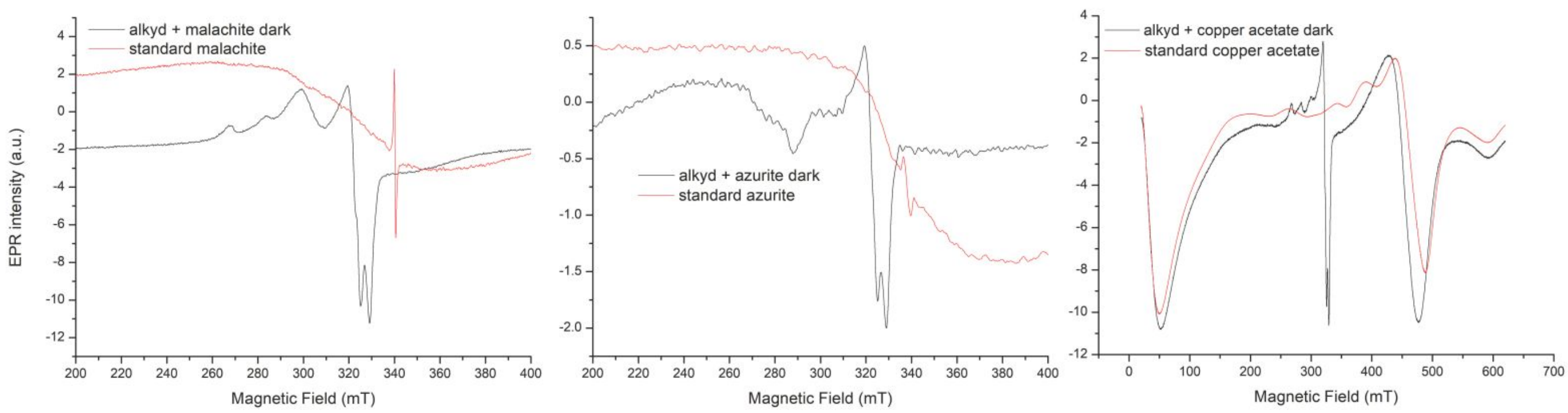

Figure S9. EPR spectra of paints submitted at museology conditions (25 ± 3 °C and 55% ± 5%) at minimum 720h and comparation with the pigment standards.

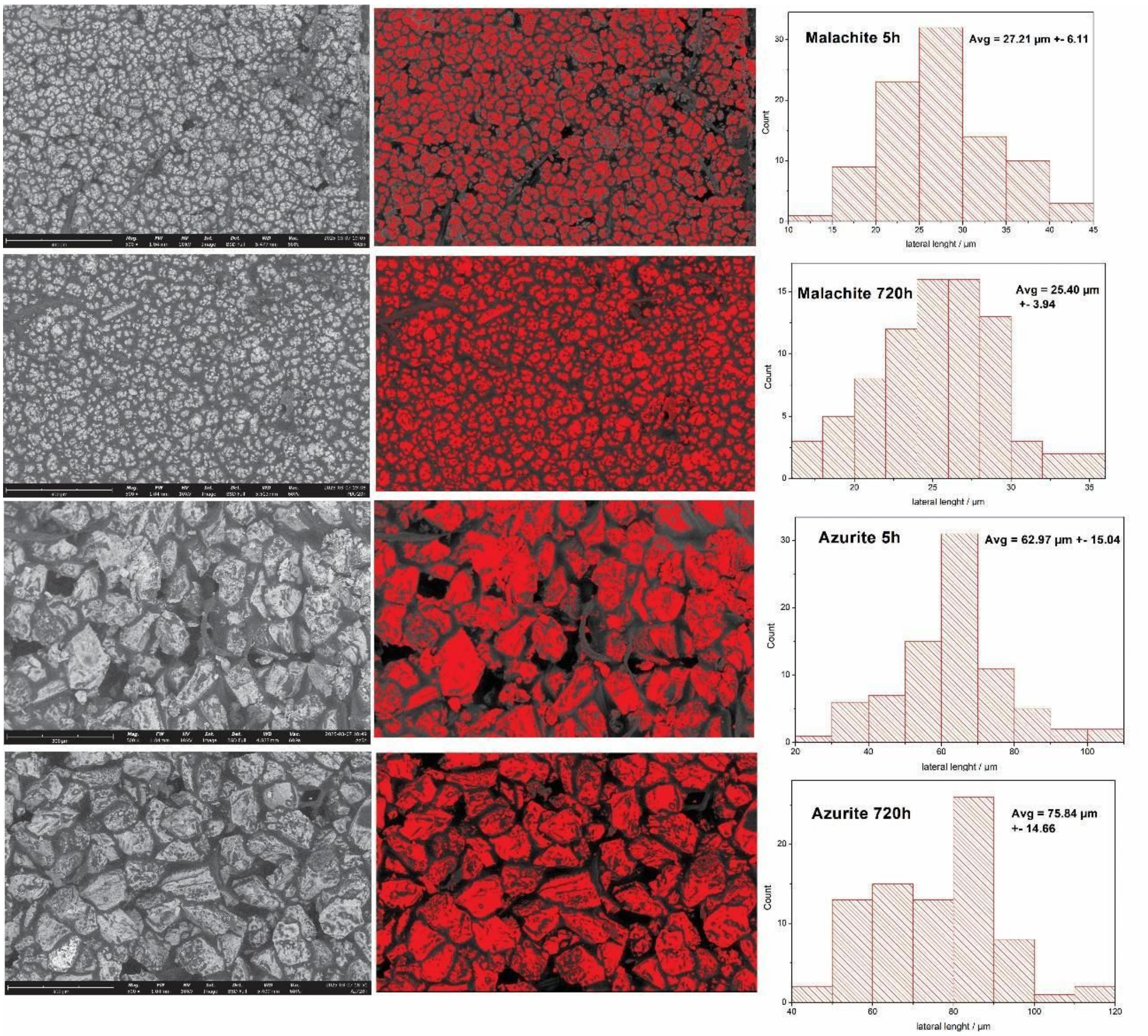

Figure S10. Micrographs of malachite and azurite samples in soybean oil alkyd resin aged for 5 and 720 hours with particle size distribution histogram. The presence of pigments in the paint layers are highlighted in red color.

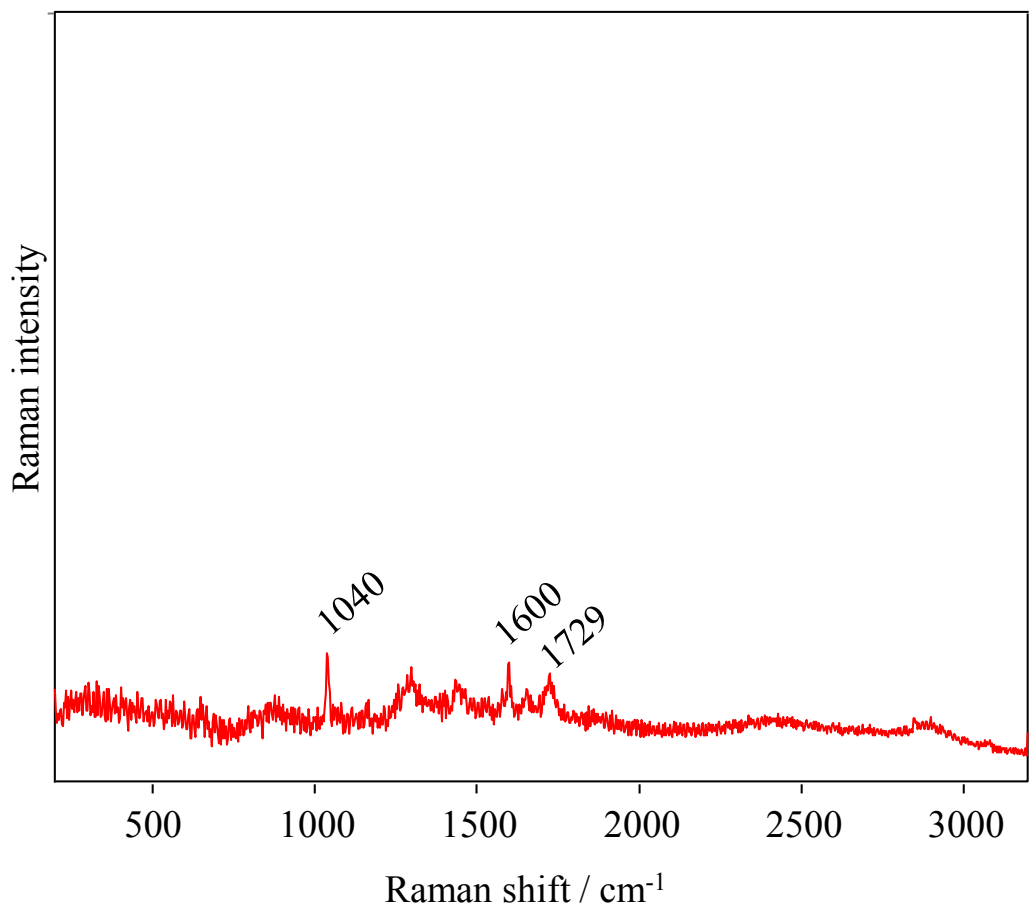

Figure S11. Raman spectra of pure soybean oil-based alkyd resin.

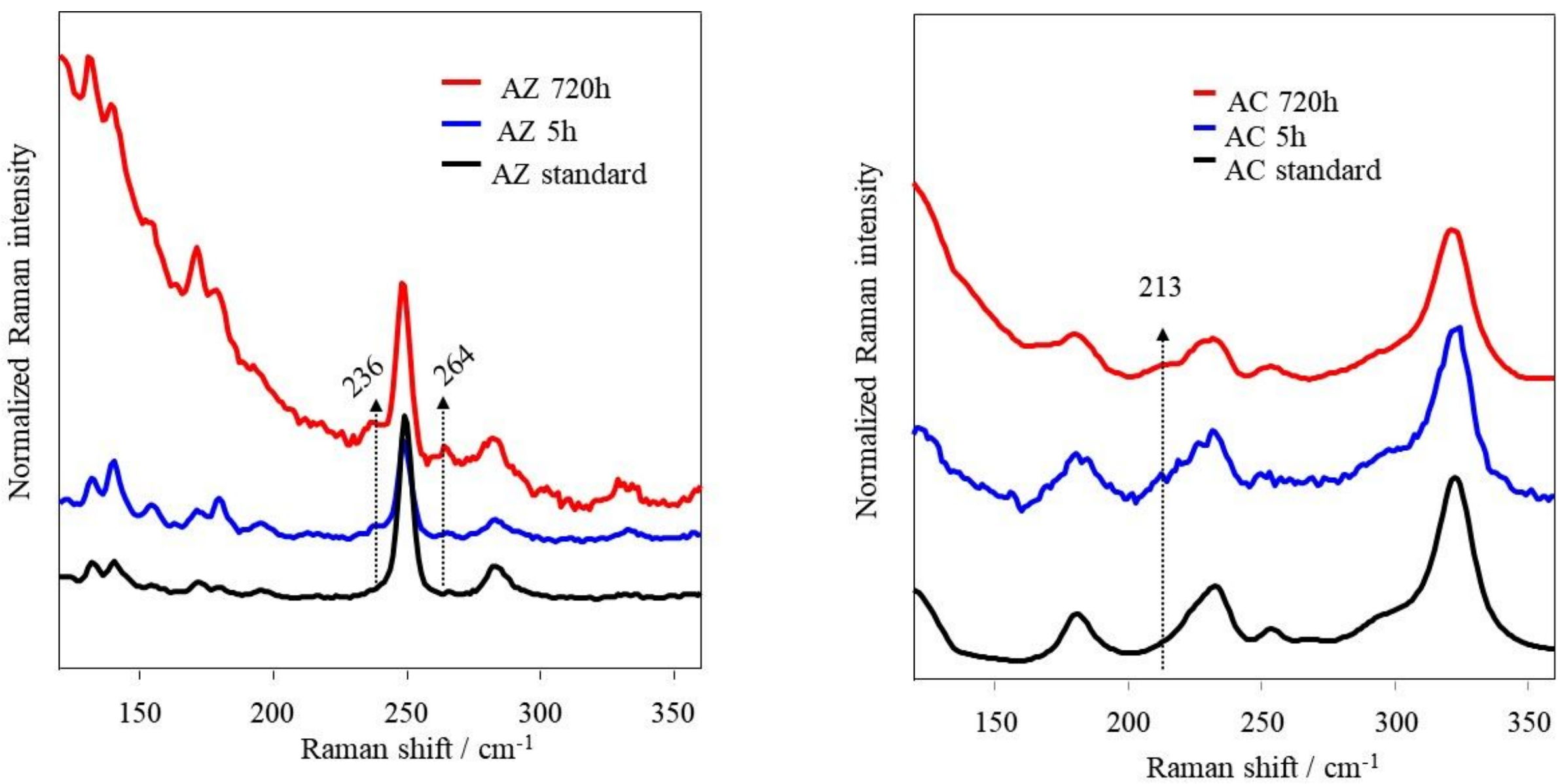

Figure S12. Raman spectra of azurite and copper acetate systems in 100-350cm^{-1} range.
